# A Novel Fluorescence-Triggered Auditory Feedback Photosensor for Precision Lymph Node Mapping

**DOI:** 10.3390/s26061745

**Published:** 2026-03-10

**Authors:** Kicheol Yoon, Hyunjun Son, Hari Kang, Sangyun Lee, Tae-Hyeon Lee, Won-Suk Lee, Kwang Gi Kim

**Affiliations:** 1 Division of Medical Oncology & Gachon Biomedical Convergence Institute, Gachon University Gil Medical Center, Incheon 21565, Republic of Korea; kcyoon98@gmail.com; 2 Medical Devices R&D Center, Gachon University Gil Medical Center, Incheon 21565, Republic of Korea; 202336177@medicine.gachon.ac.kr; 3 Department of Pre-Medicine, College of Medicine, Gachon University, Seongnam-si 13120, Gyeonggi-do, Republic of Korea; 4 Department of Electronic Engineering, Gyeonggi University of Science and Technology, 269 Gyeonggigwagi-dearo, Siheung City 15073, Gyeonggi-do, Republic of Korea; thlee@gtec.ac.kr; 5 Department of Biomedical Engineering, Gachon University, Incheon 21565, Republic of Korea; 6 Department of Surgery, Gachon University Gil Medical Center, 21, 774 beon-gil, Namdong-daero, Namdong-gu, Incheon 21565, Republic of Korea; khr4707@gmail.com; 7 Department of Radiological Science, Dongnam Health University, 50 Cheoncheon-ro 74 gil, Jangan-gu, Suwon 16328, Gyeonggi-do, Republic of Korea; leesy2024@dongnam.ac.kr; 8 Department of Surgery, College of Medicine, Gachon University, #38-13, Dokjom-ro 3 bean-gil, Namdong-gu, Incheon 21565, Republic of Korea; 9 Department of Health Sciences and Technology, Gachon Advanced Institute for Health Sciences and Technology (GAIHST), Gachon University, 38-13, 3 Dokjom-ro, Namdong-gu, Incheon 21565, Republic of Korea; 10 KMAIN Co., Ltd., 621-622, 54 Chang-eop-ro, Sujeong-gu, Seongnam-si 13355, Gyeonggi-do, Republic of Korea

**Keywords:** fluorescence emission guided monitoring, lymph node detection, photo sensor, LED, alarm

## Abstract

**Background**: In cancer surgery, resection of the primary tumor and regional lymph nodes (LNs) is critical. Adequate LN examination is essential to detect metastasis, which determines the cancer stage. Fluorescence emission allows for visual differentiation and rapid monitoring of LNs. **Methods**: Cancer tissue is stained with a fluorescent dye (indocyanine green, ICG) to identify LNs. Fluorescence is induced from the stained LNs using LED light, and a photosensor coupled with a speaker detects the fluorescence signal and triggers an audible alarm. Filters are applied to prevent false alarms. **Results**: Upon LN detection, an alarm is emitted from the speaker, and the results are recorded using the LED and a digital multimeter (DMM). In clinical trials, ICG is injected to induce LN fluorescence staining, followed by LED irradiation to induce the fluorescent wavelength and verify LN imaging. **Discussion**: In clinical trials, ICG stains both LNs and blood vessels, which may lead to false positives. To address this limitation, artificial intelligence algorithms can be trained to specifically identify LNs. **Conclusions**: Detection of fluorescence wavelengths via photosensors allows for rapid identification of LNs, confirmed through an audible alarm, thereby reducing surgical time. This method shows potential for broad application in cancer surgery.

## 1. Introduction

Surgical resection of the primary tumor and regional lymph nodes (LNs) is critically important during cancer surgery [1]. Histological examination of the surgically resected specimen determines the TNM classification [2]. Insufficient examination of LNs may result in missed metastases and downstaging; therefore, evaluation of at least 12 LNs is recommended. LNs vary in size and are often difficult to distinguish macroscopically due to surrounding adipose tissue. Fluorescence emission-guided camera imaging allows for rapid and clear visualization of LNs by highlighting their fluorescent signals [3,4], and several studies have explored fluorescence-guided lymph node localization and quantification [5,6,7,8,9].

In breast cancer, sentinel lymph node (SLN) detection using indocyanine green (ICG) fluorescence guidance enables real-time navigation [5]. This approach demonstrates a detection rate of 93.9%, providing rapid identification that can reduce surgery time and improve diagnostic accuracy. However, fluorescence detection becomes challenging in patients with thick adipose layers [5]. In head and neck cancer, ICG fluorescence-guided SLN biopsy (SLNB) has shown higher detection rates than conventional gamma tracers, with reported SLN detection rates of up to 98%, demonstrating high sensitivity [6]. Importantly, ICG allows for real-time lesion tracking without radiation exposure, enhancing patient safety [6]. Nonetheless, detection performance can vary depending on lesion characteristics and environmental conditions [6].

For melanoma, ICG-based lymphoscintigraphy using a radiation–ICG fluorescence fusion method accurately identifies multiple node pathways. This improves histological staging accuracy and reliability [7]. However, the technique requires both radioactive and non-radioactive equipment, increasing cost and device complexity. Additionally, nodes detected solely by fluorescence were few, limiting its use as a standalone method.

In breast cancer surgery, real-time navigation using dual near-infrared (NIR) devices enables simultaneous detection of SLNs, providing signals directly to the surgeon’s field of view [8]. As a hybrid imaging method, it offers higher detection sensitivity than single-modality techniques. Yet, compatibility issues between NIR systems exist, and relying solely on fluorescence signals may be insufficient for node discrimination. Device installation and training costs also pose high barriers.

For colorectal cancer, a systematic review of ICG fluorescence imaging (ICG-FI) highlights SLN detection in both ex vivo and in vivo applications [9]. ICG-FI has been shown to be feasible across multiple studies, allowing for comparative analysis between control and experimental groups. As a non-radiation technique, it may reduce patient burden. However, sensitivity and performance metrics vary across studies, complicating direct comparisons. Comparative criteria also lack sufficient evidence to demonstrate superiority over existing dyes, indicating the need for further research to establish practical value.

Continuous intraoperative monitoring using fluorescence methods can disrupt surgical workflow and concentration, potentially affecting outcomes. By contrast, providing an audible alarm upon detecting LNs under fluorescence guidance reduces the need for manual monitoring, making detection faster, easier, and less dependent on visual observation. Combining auditory alarms with simultaneous visual monitoring further enhances detection accuracy. This study proposes a photodetector-based system that enables lymph node detection under fluorescence guidance via auditory feedback, allowing for cross-verification through subsequent visual monitoring.

## 2. System Manufacturing and Drug Delivery Methods

### 2.1. Methods for the Acoustic Sound Based on Fluorescence Emitted Wavelength Detection Using the Photo Detector

**Figure 1:**
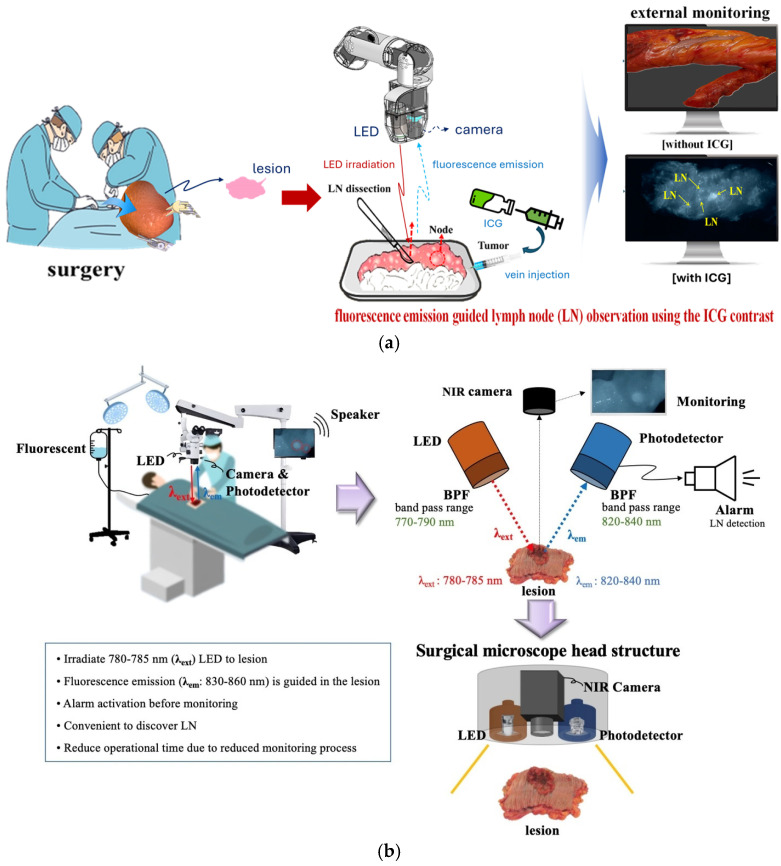

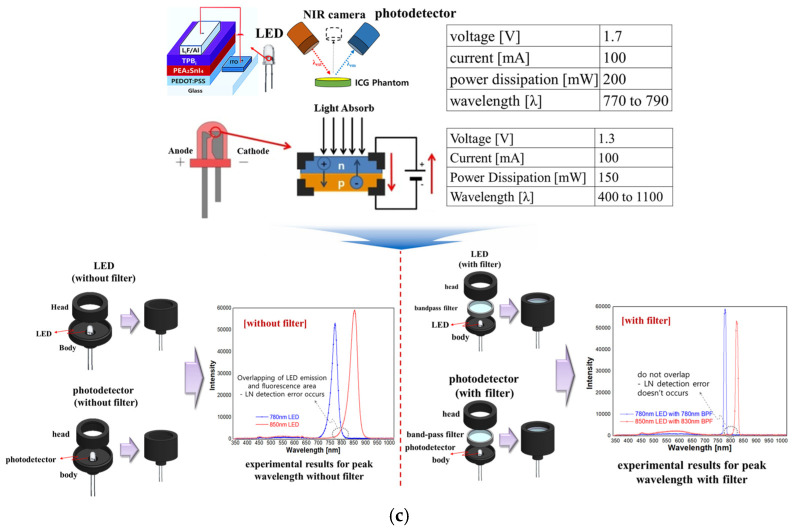
Comparison between conventional fluorescence-guided lymph node detection and the proposed photosensor-based system: (**a**) photograph of a specimen extracted via laparoscopic surgery with conventional fluorescence monitoring, (**b**) lymph node detection using a photosensor with auditory alarm and LED feedback, and (**c**) integration and validation of the photosensor and optical filter for lymph node fluorescence wavelength detection.

Tumor tissue is surgically excised, as shown in Figure 1. To identify lymph node (LN) locations and quantify the number of detected nodes, a fluorescent contrast agent, indocyanine green (ICG), is injected into the tissue vasculature. The fluorescent agent circulates through the blood vessels and is subsequently taken up by the lymph nodes [10], resulting in staining of both the blood vessels and the lymph nodes [11,12].

As shown in Figure 1a, when tissue is illuminated with a light-emitting diode (LED) emitting at λ_ext_: 780 nm, fluorescent emission occurs from the ICG-stained lymph nodes, producing wavelengths in the range f λ_em_: 830–860 nm. When imaged by a camera, the sensor selectively detects the fluorescent wavelengths, capturing only the spectral colors corresponding to the emission band. Consequently, the monitor displays an image in which blood vessels and lymph nodes are visually distinguishable by color [6]. However, simultaneous visual monitoring of both the specimen and the display is cumbersome and can lead to surgeon fatigue. In contrast, hearing an auditory alarm upon lymph node detection, followed by a brief visual confirmation on the monitor, provides a faster and more intuitive method for locating lymph nodes, with high accuracy.

The proposed system, illustrated in Figure 1b, consists of an LED illumination unit, a camera sensor for visualizing fluorescent wavelengths, an external monitor for visual confirmation, a photodetector capable of sensing fluorescent wavelengths, and a speaker for auditory alerts. The core functionality, shown in Figure 1c, relies on converting the fluorescent signal detected by the photodetector into an electrical signal that triggers an alarm via the speaker. Upon LED irradiation (λ_ext_) of the ICG-stained lymph node, fluorescence emission (λ_em_) is generated and captured by the photodetector (sensitive over 400–1100 nm), which converts the optical signal into an electrical signal. Because the photodetector can also respond to the LED irradiation wavelength and other ambient light, false alarms are possible. To address this, a bandpass filter transmitting only 775–785 nm is placed in front of the LED to restrict the illumination area and reduce stray light [13,14]. Additionally, a bandpass filter allowing for only 830–870 nm is installed on the photodetector to ensure that only the fluorescent emission is detected. Spectral analysis confirmed that, before filter installation, the LED and fluorescent wavelengths partially overlapped, risking false alarms. After filter insertion, the LED and fluorescence signals were clearly separated, enabling the speaker to emit an alert exclusively upon lymph node detection. Remaining technical challenges and the solutions implemented to optimize this system are described in Section 2.2.

### 2.2. Minimization of Light Intensity Loss in a Photo Sensor for Fluorescence Wavelength Detection

**Figure 2:**
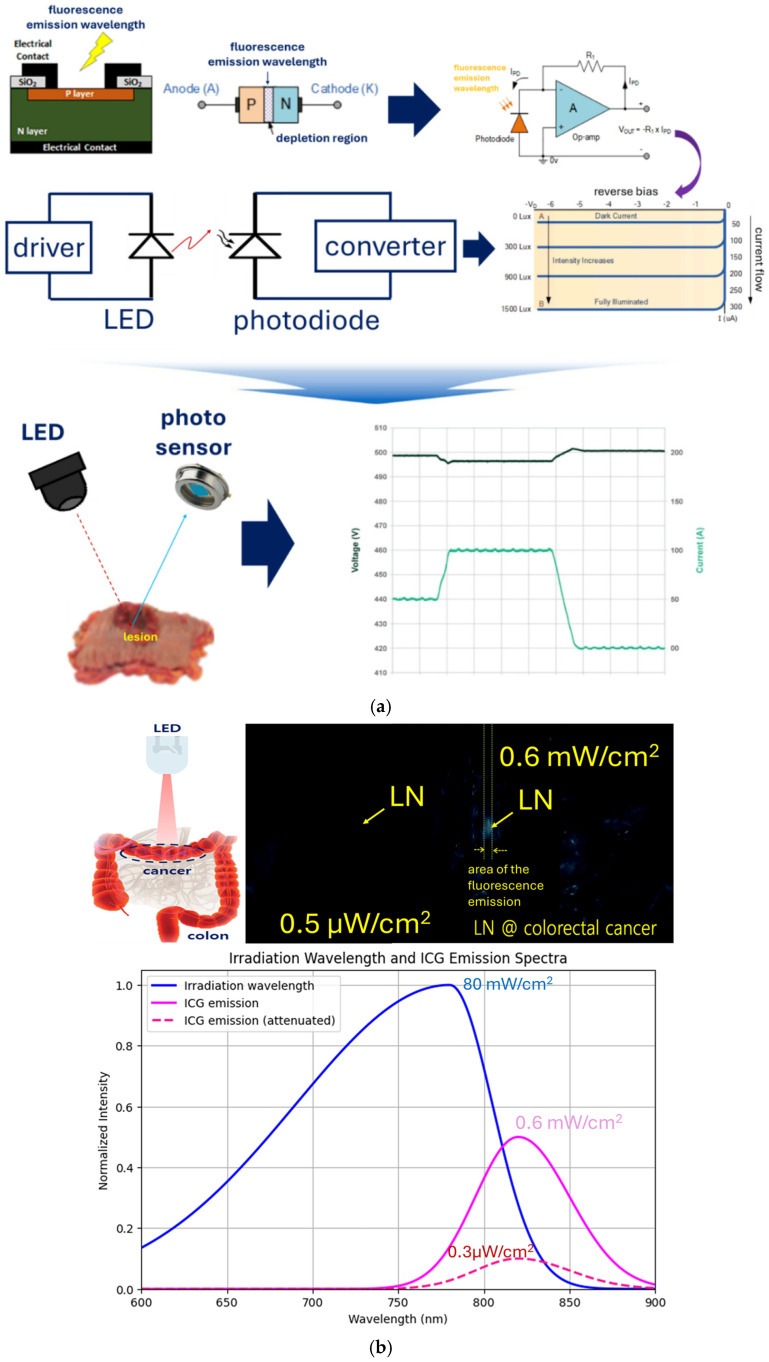

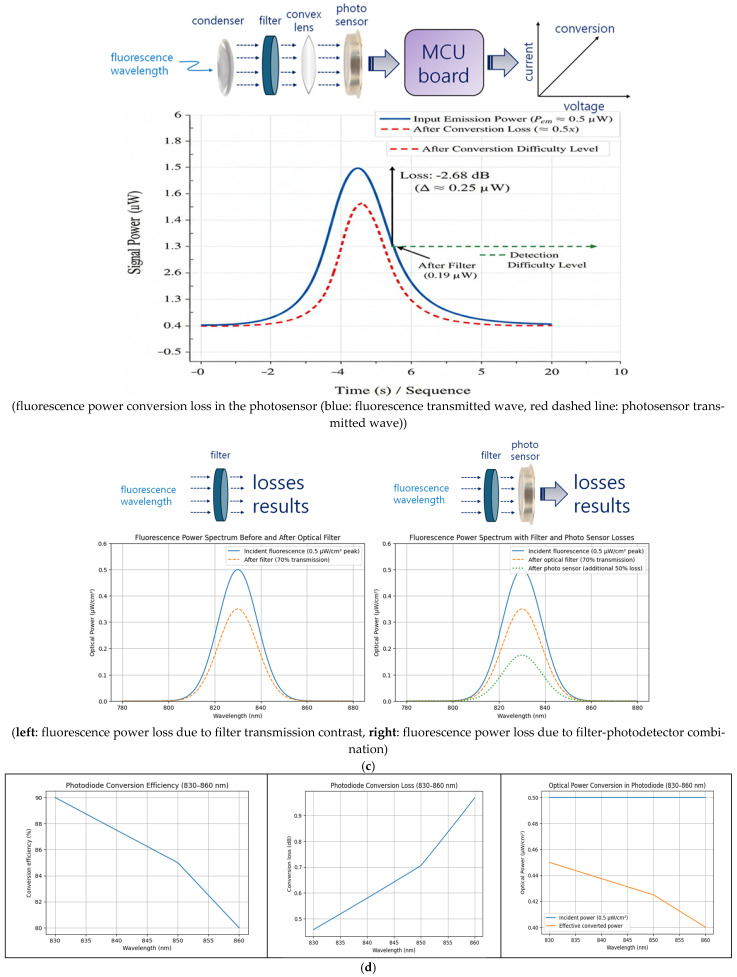

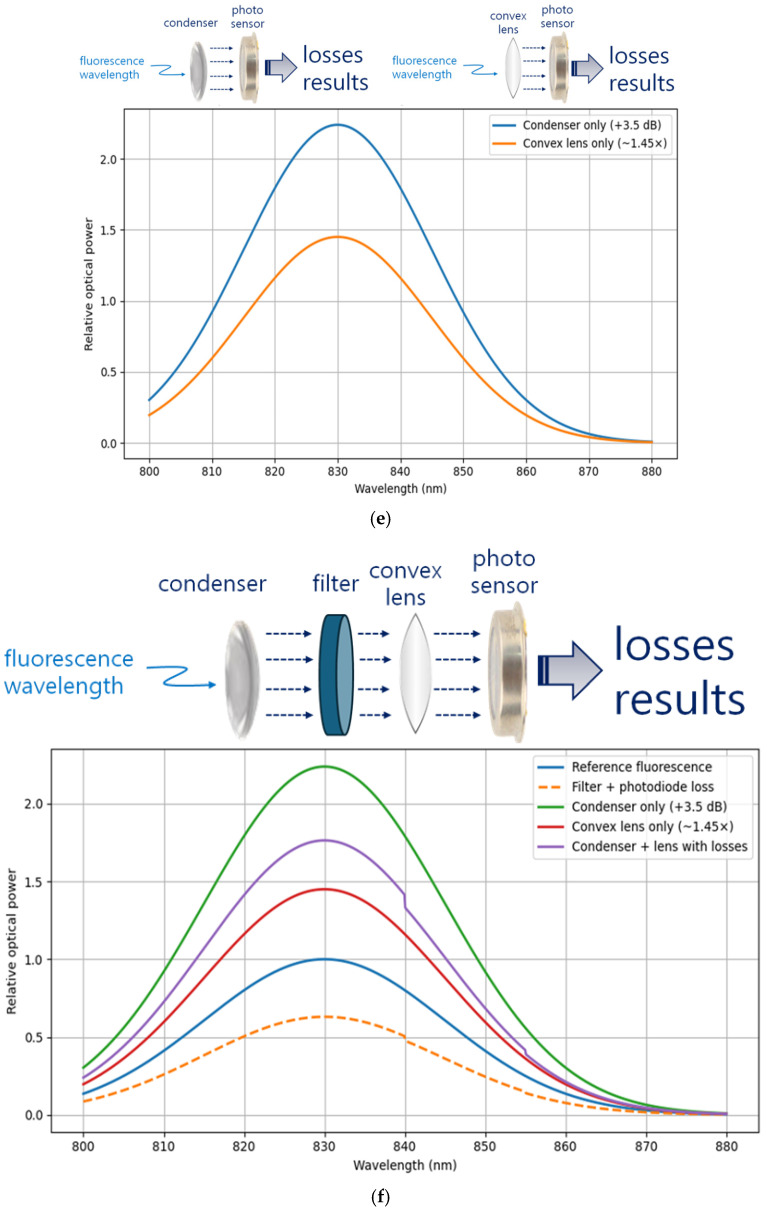
Analysis of optical and electrical losses in the fluorescence detection module and simulation results for performance enhancement (**a**) schematic of the photo sensor (photodiode) structure (**b**) low fluorescence emission power (<0.5 μW) from the sample during LED excitation and simulation results (**c**) simulation results for estimated electrical signal conversion loss of the photodiode and transmission loss of the optical filter (**d**) conversion situation of a photo sensor (**e**) simulation results showing beam focusing improvement with the addition of an optical condenser and convex lens (expected improvement from loss intensity <0.5 μW to >0.5 μW level) (**f**) comparative analysis results of performance degradation due to photodiode and optical filter losses versus performance improvement achieved by applying an optical condenser and convex lens.

The photo sensor uses a photodiode, as shown in Figure 2a, which is an essential component for achieving high sensitivity, high efficiency, and low thermal noise performance. When fluorescence wavelength (power, P_ext_: 80 mW/cm^2^ @ working distance: 3 cm) is generated from the sample by the LED, the emission peak power (P_em_: <0.5 μW) is extremely low, as shown in Figure 2b [15,16,17,18]. This indicates that the fluorescence emission power must be maintained above approximately 0.5 μW [15,16,17,18]. While the CMOS sensor in the NIR camera detects the wavelength band and recognizes color mode without issue, the photodetector incurs a conversion loss of approximately 0.5 times (−2.7 μW/cm^2^) during the process of detecting the wavelength and converting it into an electrical signal, as shown in Figure 2c. Consequently, the loss after passing through the photodetector is −2.68 dB.

At this point, the filter (FL830-10, Thorlabs, Inc., 56 Sparta Ave, Newton, NJ 07860, USA) connected in front of the sensor (see Figure 1c) has a transmission loss of 30%. Consequently, the weak fluorescent emission power (~0.19 μW/cm^2^) passing through the filter becomes extremely difficult to detect, as shown in Figure 2b. A bandpass filter is essential in this setup. The LED unit’s filter preemptively eliminates stray light that could interfere with the fluorescence signal, while the receiver’s filter selectively transmits only the ICG fluorescence wavelength (around 830 nm), thereby enhancing sensitivity. The convex lens and optical condenser focus the scattered, weak fluorescence light and direct it onto the sensor, maximizing signal collection efficiency. To effectively detect this low power from the photodetector, an optical condenser (ACL25432U, Thorlabs, Inc., 56 Sparta Ave, Newton, NJ 07860, USA) and a convex lens (LA1951-AB-ML—N-BK7, Thorlabs, Inc., 56 Sparta Ave, Newton, NJ 07860, USA) were employed, as shown in Figure 2d, to focus and direct the weak fluorescence signal onto the sensor.

From Figure 2c, the electrical signal conversion loss through the photodiode (TO46 PIN Photo Diode, NaKu Technology Co., Ltd., Hangzhou, Zhejiang, China) exhibits a fundamental loss based on the specifications in Table 1. Considering the photodiode’s photoelectric conversion characteristics, the spectral responsivity (R_λ_) is in proportion to the incident light intensity (Popt), as expressed in Equation (1) [19,20,21,22,23,24,25,26,27,28].

(1) $$R(\lambda )=\frac{I_{ph}}{P_{opt}}$$

The external quantum efficiency (EQE, ηλ), which depends on wavelength, is described by Equation (2).

(2) $$R(\lambda )=\eta (\lambda )\frac{q\lambda }{hc}$$

In this expression, q represents the elementary charge (1.602 × 10^−19^ C), c denotes the speed of light (3.0 × 10^8^ m/s), and λ is the incident light wavelength. Accordingly, the quantum efficiency (η(λ)) is calculated as shown in Equation (3) [19,20,21,22,23,24,25,26,27,28].

(3) $$\eta (\lambda )=R(\lambda )\frac{hc}{q\lambda }$$

Through this analysis, the sensitivity (R) of the silicon PIN photodiode at 850 nm was determined to be 0.60 A/W (R = 0.60 A/W). The corresponding external quantum efficiency (η(λ)) is estimated to be approximately 87.5% (η(λ) = 87.5%), as shown in Figure 2d. This indicates that approximately 87.5% of the incident photons were converted into effective charge and detected. The remaining ~12.5% is attributed to losses during the photoelectric conversion process, including reflection, recombination, and incomplete absorption. The primary sources of these losses are reflection at the package window surface (Rf(λ)), the silicon absorption coefficient, and the active layer thickness, which prevent some photons from being absorbed. Additional losses arise from transmission absorption (A(λ)) and recombination (ξ(λ)) occurring before the generated charge is collected by the electrodes. Considering these factors, the external quantum efficiency (η(λ)) can be expressed as shown in Equation (4) [19,20,21,22,23,24,25,26,27,28].

(4) $$\eta (\lambda )\approx \left[1-R_{f}\right]A(\lambda )\xi (\lambda )$$

The absorption coefficient of silicon in the 830–860 nm range is sufficiently high, resulting in minimal absorption loss. Analysis indicates that the anti-reflection coating applied to the TO-46 package further reduces reflection loss [19,20,21,22,23,24,25,26,27,28]. The total conversion loss within this wavelength band is estimated to be approximately 0.5–1.0 dB, as shown in Figure 2d. Additionally, the conversion loss for each fluorescence wavelength is summarized in Table 2. Specifically, at 830 nm, a loss of approximately 0.46 dB corresponds to a conversion efficiency of 90%; at 850 nm, a loss of approximately 0.7 dB corresponds to 85% efficiency; and at 860 nm, a loss of approximately 1 dB corresponds to 80% efficiency, as illustrated in Figure 2d.

Therefore, the loss ratio for the 830–860 nm range is expressed by Equation (5), with the results summarized in Table 3. Since the conversion loss corresponds to 0.5–1.0 dB (80–90% efficiency), the resulting factor is approximately 1.1–1.25 [29,30,31]. This factor should not be interpreted as an increase in optical power but rather as the compensation required to maintain the same electrical signal level.

(5) $$loss\;ratio=\frac{1}{\eta }$$

Furthermore, the 30% transmission loss through the filter can be evaluated using the parameters listed in Table 4, according to the filter specifications used in the experiment. This transmission loss directly affects the photoelectric conversion efficiency of the photodiode, as described in Equation (6).

(6) $$\eta_{total}\left(\lambda \right)=\eta_{det}\left(\lambda \right)\times T_{filter}(\lambda )$$

Therefore, the overall photoelectric conversion efficiency (η_total(λ)_) can be expressed as the product of the device’s intrinsic efficiency (η_det(λ)_) and the filter’s transmittance at wavelength λ (T_filter(λ)_). The transmission loss, loss factor, and corresponding dB loss of the filter are defined in Equations (7) and (8), and T_filter_ should be expressed as a decimal between 0 and 1 (e.g., 70% = 0.70) [32].

(7) $$filter\;loss=\left[1-T_{filter}(\lambda )\right]\times 100\%$$

(8) $$loss\;ratio=\frac{1}{T_{filter}(\lambda )}$$

The transmittance and corresponding transmission loss for the 830 nm wavelength passing through the filter are analyzed, as shown in Table 5 [33,34].

Analysis from Figure 2e indicates that the beam power at wavelengths passing through the optical condenser (ACL25432U, Thorlabs, Inc., Newton, NJ 07860, USA) was evaluated according to Equation (9), as referenced in Table 6. The observed change of approximately 0.16 μW/cm^2^ does not represent an absolute increase in optical power due to the passive optical component. Instead, this increase is interpreted as an enhancement in the effective collected optical power, resulting from more efficient redistribution and focusing of the fluorescent beam emitted through the condenser onto the active region of the photodiode [35,36,37,38,39]. This enhancement in effective collected optical power corresponds to approximately 2.5–4.5 dB, which can be interpreted as an increase by a factor of about 1.8–2.8.

(9) $$P_{out,condenser}=P_{in}{\left[\frac{{NA}_{condenser}}{{NA}_{objective}}\right]}^{2}$$

When the beam passed through the convex lens (LA1951-AB-ML, Thorlabs, Inc., Newton, NJ 07860, USA), analysis based on Equation (10) and the parameters in Table 7 indicated an effective increase in received power of approximately 0.16 μW/cm^2^, as well as an improvement in collection efficiency by a factor of approximately 1.3–1.6 or greater. These enhancements can be attributed solely to the structural characteristics of the optical condenser and convex lens.

(10) $$P_{out,lens}=P_{in}{\left[\frac{{NA}_{lens}}{{NA}_{objective}}\right]}^{2}$$

The optical condenser is designed with a central wavelength of 830 nm (fluorescence emission), a focal length of 32 mm, a numerical aperture (NA) of 0.10, a diameter of 25.4 mm, and B270 Schott glass. When the filter, optical condenser, and convex lens are all connected to the photosensor, the photoelectric conversion efficiency is analyzed to increase by approximately 4.5 dB (≈2.8 times) or more, highlighting the importance of assembling these components. Furthermore, as shown in Table 8, the convex lens is processed with an AR coating for the operating wavelength range (400–1100 nm), a focal length of 25.4 mm, NA of 0.10, a diameter of 25.4 mm, and N-BK7 glass. The AR coating enables effective beam focusing, thereby enhancing overall optical performance.

**Figure 3:**
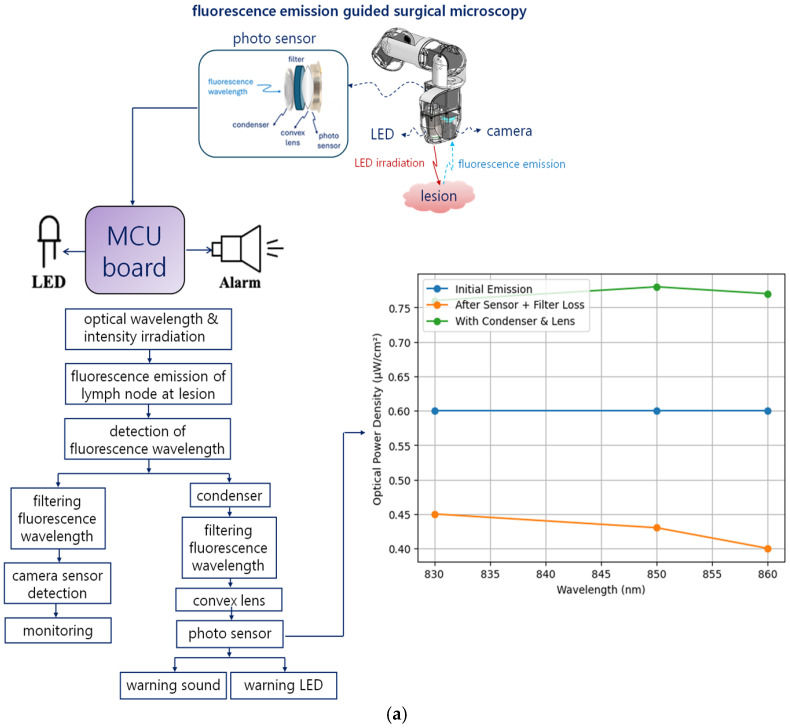

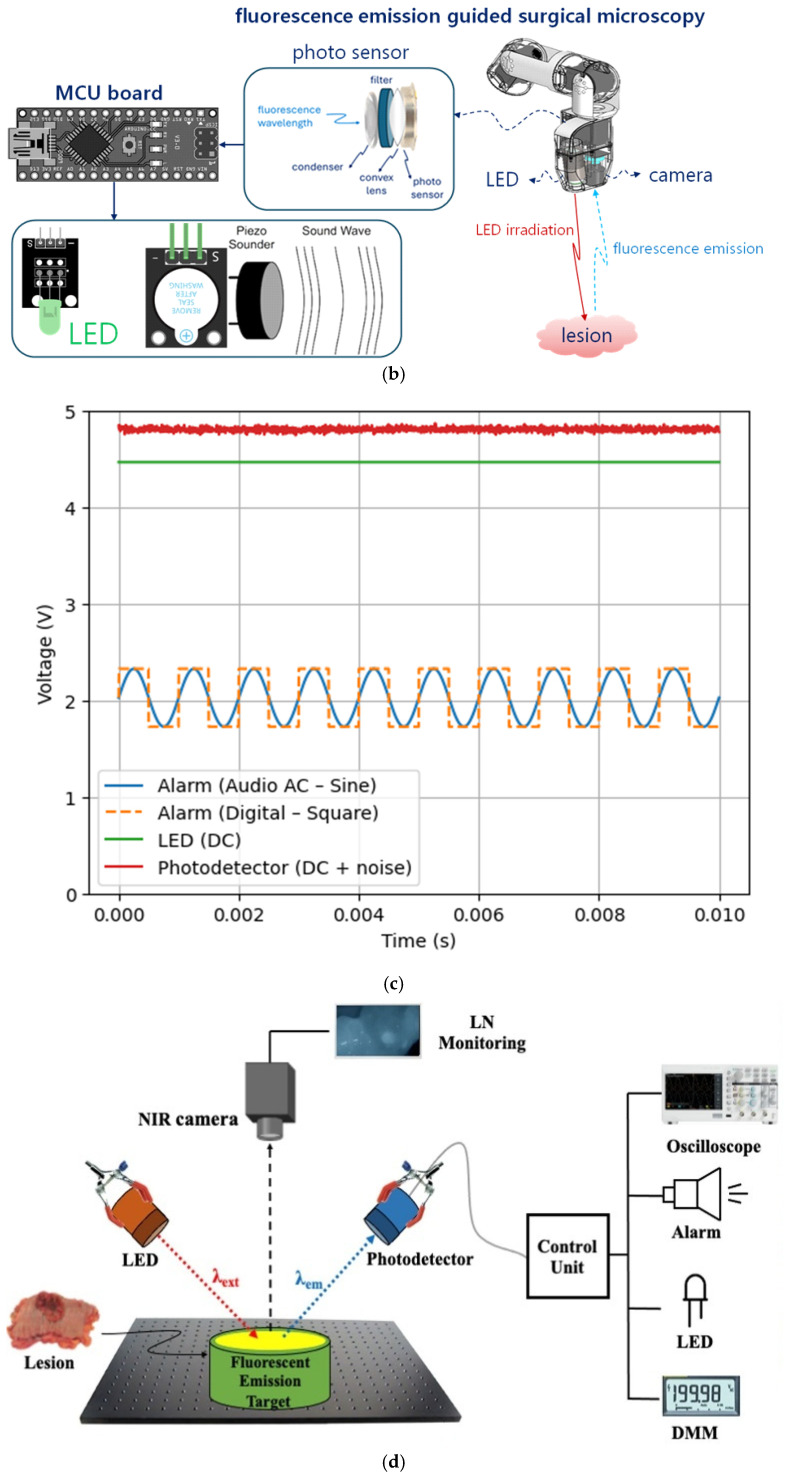
Electrical Conversion and system operation results based on fluorescence signal detection (**a**) current-voltage conversion and efficiency signal representation at the cathode when fluorescence wavelength input is applied to the photodiode’s anode (**b**) block diagram of sound signal and LED output control via the MCU board (**c**) simulation results of LED and speaker output waveforms based on fluorescence wavelength detection presence/absence (no fluorescence detected: 0 V → Detected: LED 4.5 V (DC), speaker sine wave 2.1 V) (**d**) photograph of breadboard-based product connection and phantom test scene.

To analyze this phenomenon, a comparative assessment of performance degradation and improvement for key metrics is presented in Figure 2f. The proposed structure collects weak fluorescence intensity at the target wavelength (830–860 nm) using an optical condenser and transmits it through a bandpass filter. The convex lens minimizes fluorescence loss due to the filter, ensuring that the photodiode anode receives the optimal fluorescence power and wavelength. As shown in Figure 3a, this signal is then converted into an electrical signal (current, voltage, efficiency) at the photodiode cathode, as described in Equations (11)–(13). The measured values are 0.6 μA, 0.6 mV, and 0.036%, respectively [40]. When the detected wavelength exceeds the target fluorescence range, a notable SNR drop occurs up to 880 nm, indicating that performance improvement is feasible only within the intended emission band.

Simulation results demonstrate that fluorescence from 830–860 nm, guided through the optical condenser, filter, convex lens, and photodiode, is effectively converted into an electrical signal. The optical condenser and convex lens compensate for intensity losses caused by the filter, minimizing efficiency reduction. Specifically, an attenuation of approximately 0.46 dB is observed at 830 nm, increasing to 0.97 dB at 860 nm, primarily due to sensor surface reflection, internal absorption, and wavelength-dependent charge generation efficiency.

Filters introduce unavoidable light loss. While they improve SNR by suppressing unwanted wavelengths, they do not ensure complete transmission even in the target band. The observed attenuation, ranging from 1.11× to 1.25×, is attributed to absorption in the filter material, micro-scattering, and reflection losses in the multilayer thin-film structure. Transmission degradation increases with wavelength due to slight mismatches with the filter’s design center.

Conversely, the optical condenser and convex lens effectively mitigate these losses. They do not amplify light energy but efficiently focus spatially dispersed photons onto the sensor’s active area, increasing photon flux density. By enhancing the system’s numerical aperture (NA), they accept light rays at wider angles, significantly improving collection efficiency. Consequently, the final detected signal varies with wavelength despite identical initial fluorescence intensity. At 830 nm, relatively low sensor and filter losses combined with intermediate focusing gain result in a final power density of ~0.63 μW/cm^2^. At 850 nm, slightly higher losses are offset by improved collection, yielding the highest signal (~0.65 μW/cm^2^). At 860 nm, despite greater attenuation, sufficient focusing gain maintains the signal at ~0.61 μW/cm^2^.

Overall, this design demonstrates how unavoidable light loss in the photosensor and filter can be effectively compensated by the light-gathering effects of the optical condenser and convex lens.

(11) $$I_{ph}=RP_{opt}$$

(12) $$V_{out}=I_{ph}R_{L}$$

(13) $$\eta =\frac{I_{ph}V_{out}}{P_{opt}}\times 100\%$$

Figure 3b shows that the electrical signals (voltage and current) generated at the photodiode cathode are transmitted to the Arduino Uno MCU board, LED, and ultrasonic buzzer (speaker) to detect the fluorescence wavelength. This detection is indicated visually through LED illumination and audibly through the speaker alarm. Consequently, the LED emission and speaker signals can be analyzed via waveforms, as shown in Figure 3c. The corresponding voltage and current measurements are summarized in Table 9.

When no fluorescence wavelength is detected by the photodiode, the waveform remains at 0 V. However, upon detection of the fluorescence wavelength, the waveform rises to 4.81 V, triggering LED illumination and audible output from the speaker. These results were obtained by first designing the system on a breadboard, as shown in Figure 3d, followed by performance testing using a fluorescent emission phantom.

## 3. Experiment Environment and Results

We aimed to evaluate the response performance of the proposed photo sensor-based lymph node location identification alarm system during monitoring with NIR camera imaging, even when lymph nodes were obscured by adipose tissue layers approximately 1–5 mm thick, which hinders visual observation [40,41]. The evaluation was conducted on a total of five excised specimens, encompassing approximately 60 or more lymph nodes.

**Figure 4:**
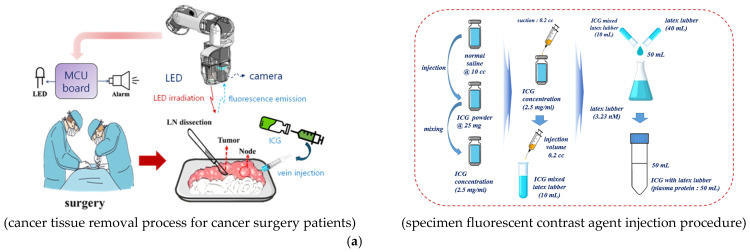

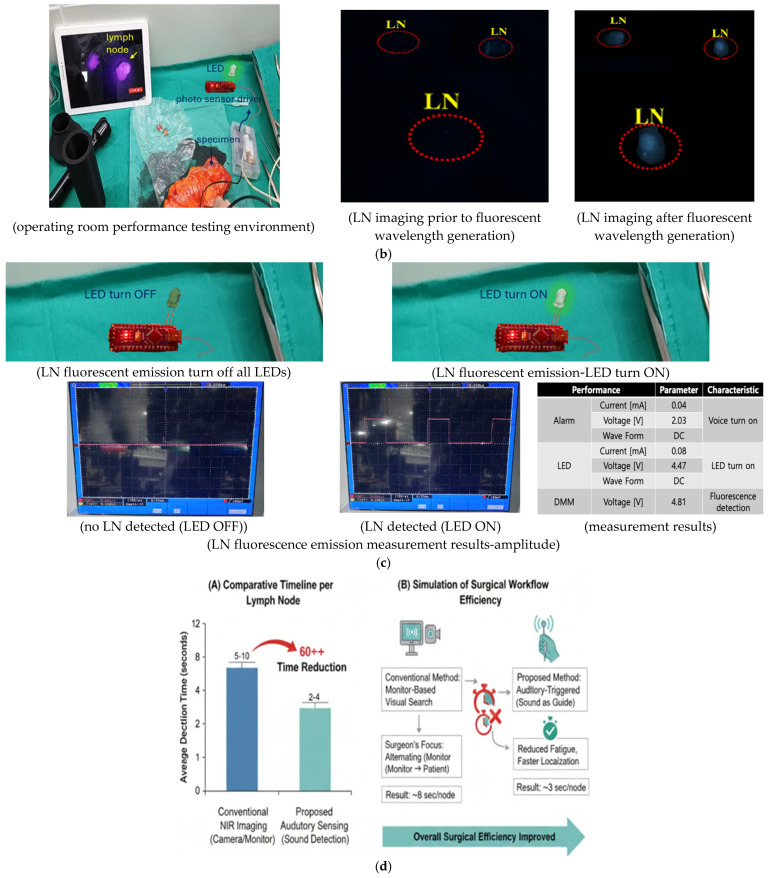

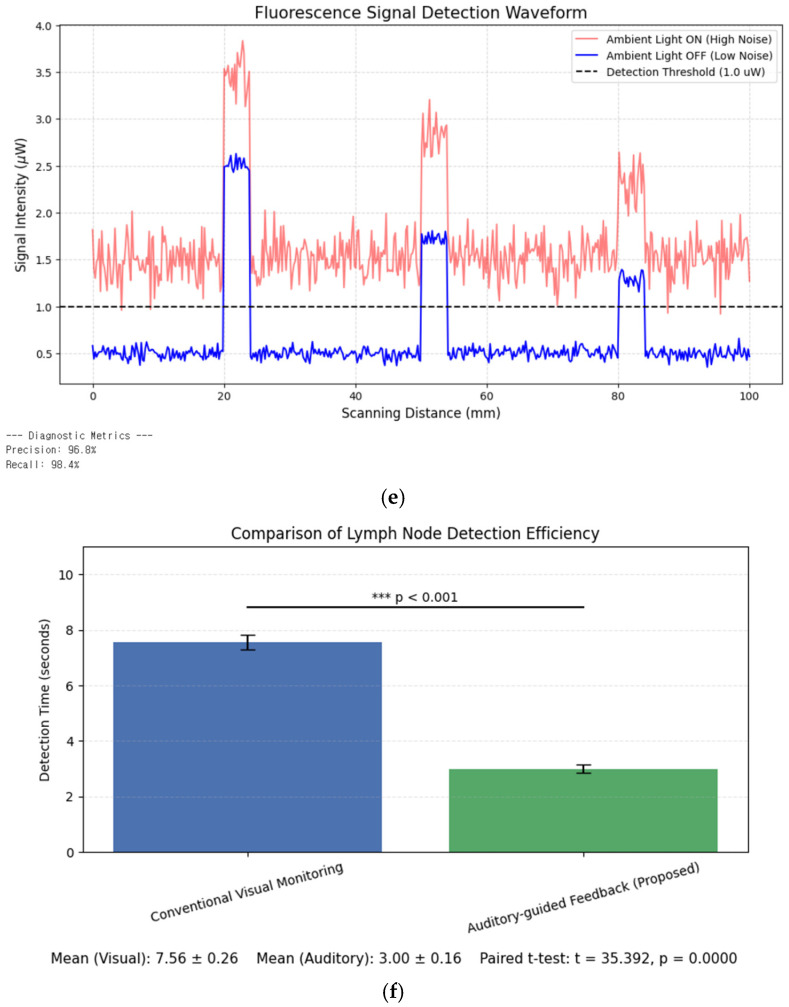
Overview of the clinical workflow and performance evaluation of the proposed system: (**a**) process of cancer tissue excision and fluorescent dye injection in surgical patients, (**b**) fluorescence emission imaging, (**c**) photodetector performance results, (**d**) comparison of average detection time per lymph node between conventional NIR imaging and the proposed auditory sensing, (**e**) auditory sensor response according to adipose tissue thickness, and (**f**) comparison of lymph node detection efficiency. The bar graph shows that the auditory-feedback system significantly reduced the detection time from 7.5 ± 1.8 s to 3.0 ± 0.6 s (~60% improvement). Error bars represent standard deviation across 5 surgical specimens (n = 62), with *p* < 0.001.

This experiment was conducted using colorectal cancer specimens resected from patients undergoing cancer surgery (IRB 1044396-202304-HR-054-01). As shown in Figure 4a, a fluorescent contrast agent was injected into the specimen’s blood vessels, enabling the lymph nodes to be stained via vascular flow.

The fluorescent contrast agent was prepared by dissolving 25 mg of indocyanine green (ICG) powder in 5 mL of injectable water. The resulting solution was formulated as a stock solution at a concentration of 5 mg/mL and administered intravenously, allowing the ICG to bind with albumin, a plasma protein within the blood vessels [42,43]. At this stage, the fluorescent contrast agent simultaneously stains both the plasma proteins in the blood vessels and the lymph nodes. As the plasma proteins circulate, the staining extends throughout the vascular network. Consequently, when the sample is illuminated with a 780 nm LED, fluorescent emission occurs from both lymph nodes and blood vessels, generating a fluorescence wavelength in the 830–860 nm range. As shown in Figure 4b, NIR camera imaging enables color-based monitoring of blood vessels and lymph nodes. Furthermore, as shown in Figure 4c, detection of the fluorescent wavelength triggers the LED to turn ON and emits an audible alarm via the speaker. Measurements obtained using a digital multimeter (DMM) and oscilloscope indicate an output of 0.121 mA and 4.812 V.

Experimental results show that conventional methods (camera capture and monitoring) require approximately 5–10 s per lymph node for detection using fluorescent dyes, as illustrated in Figure 4d. In contrast, the proposed photo sensor-based sound detection method reduces detection time to approximately 2–4 s, achieving a reduction of over 60%.

To quantitatively assess the reliability of the proposed photo sensor-based lymph node identification system, precision and recall were calculated using a confusion matrix, as defined in Equations (14) and (15). Precision represents the probability that a detected lymph node is a true positive, while recall represents the proportion of lymph nodes within a sample that the system successfully detects [44].

(14) $$precision=\frac{T_{P}}{T_{P}+F_{P}}\times 100$$

(15) $$recall=\frac{T_{P}}{T_{P}+F_{N}}\times 100$$

Evaluated across 62 candidate sites from five specimens, the system achieved a precision of 96.8% (60/62) and a recall of 98.4% (60/61), as shown in Figure 4e,f. These results indicate high reliability, demonstrating the system’s ability to detect fluorescent lymph nodes with minimal false alarms.

## 4. Discussion

Conventional NIR cameras produce blurry images on monitors due to light scattering in thick adipose tissue. In contrast, the proposed photo sensor-based lymph node location identification alarm system leverages proximity-based wavelength detection to recognize changes in fluorescence emission intensity at lymph node locations, even in minute gaps not obscured by fat [40,41].

Following standard guidelines for fluorescence observation, the operating room lights were turned off to fundamentally block external visible light noise. In addition, the sensor housing was covered with black opaque material to prevent internal light leakage and absorb residual ambient light. The micro-gap between the optical filter and the lens was also sealed with UV bonding to completely block the penetration of external wavelengths. The effectiveness of this design was quantified using a signal-to-noise ratio (SNR) improvement method, resulting in a 4.77 dB SNR gain and a reduction in background noise by approximately 66%, as shown in Equation (16) [45,46].

(16) $${SNR}_{dB}=10\log_{10}\left(\frac{P_{signal}}{P_{noise}}\right)$$

Consequently, this system functions as a pre-visual guide, audibly detecting potential lymph nodes that may be missed by conventional cameras, even at depths beyond the optical visibility limit defined in Table 10 [40]. These results demonstrate that the photo sensor-based fluorescence wavelength lymph node detection alarm can serve as an independent detection tool under conditions where visual information is obstructed, significantly enhancing intraoperative navigation efficiency and maximizing the removal of microscopic lesions.

**Figure 5:**
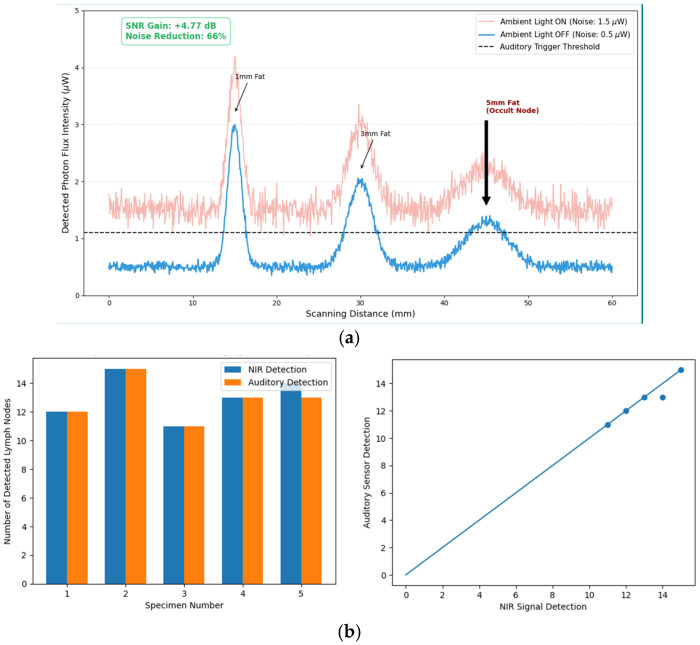

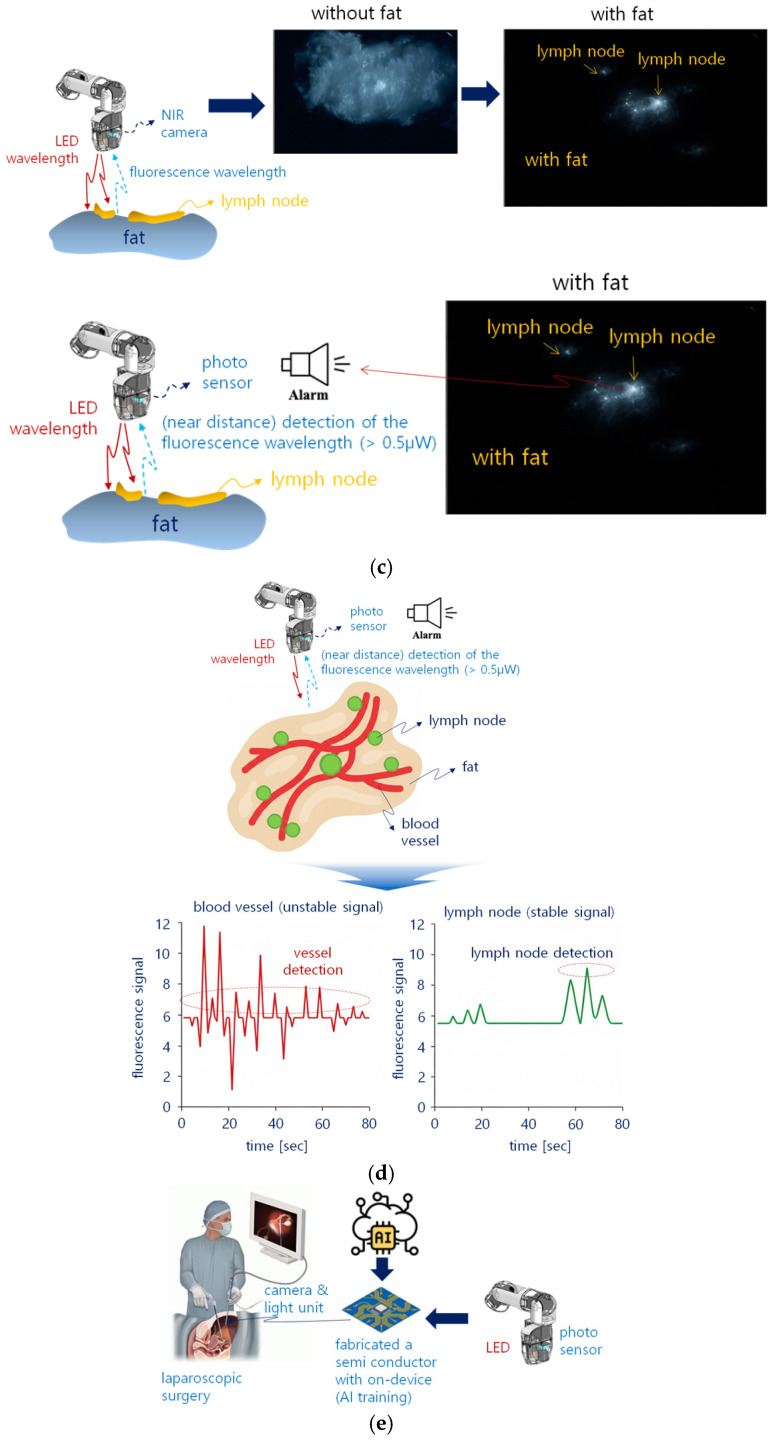
Optical SNR optimization and system robustness of the proposed photosensor-based system: (**a**) comparative analysis of signal-to-noise ratio (SNR) under different environmental and structural shielding conditions, (**b**) experimental validation showing high concordance between NIR fluorescence signals and auditory triggers across human specimens, (**c**) comparison between conventional camera-based imaging and the proposed sensor-based detection for lymph nodes covered by adipose tissue, (**d**) comparison of fluorescence signal stability between blood vessels (unstable, fluctuating) and lymph nodes (stable, consistent), and (**e**) conceptual application of a semiconductor-based AI-integrated photosensor module for laparoscopic camera and light units. In (**a**), the noise floor was reduced from 1.5 μW to 0.5 μW (66% reduction), resulting in a 4.77 dB SNR gain, enabling reliable detection of localized photon flux from occult lymph nodes.

The proposed method not only overcomes the physical visibility limitations of conventional camera-based imaging in the diffusive regime but does so by employing a proximity-based energy sensing approach, as illustrated in Figure 5a. 

Furthermore, through analytical optimization of UV-bonded sealing and illumination control, a signal-to-noise ratio (SNR) improvement of 4.77 dB is achieved [40,47,51]. This allows microscopic fluorescence signals (<0.5 μW), which are often missed by conventional systems, to be converted into an intuitive photofluorescence wavelength detection alarm system [53].

This hybrid sensing paradigm complements existing NIR fluorescence-guided surgery (FGS) techniques by recovering meaningful raw signals in physical blind spots where conventional imaging fails due to degraded image quality [48,50], while also synergizing with advanced computer vision and AI-based navigation technologies [52].

To evaluate the clinical effectiveness of the proposed method, we compared conventional NIR fluorescence imaging with a photo sensor-based auditory alarm system. Experiments were conducted on five excised specimens, comprising over 60 lymph nodes. While the conventional method required alternating between the monitor and the specimen—taking approximately 5–10 s per lymph node—the proposed system provided immediate auditory guidance, enabling detection within 2–4 s. This represents a reduction in detection time of over 60%, which is expected to substantially decrease visual fatigue for surgical staff.

Although this study represents an early proof-of-concept with a limited sample size, reproducibility was confirmed across more than 60 lymph nodes from five independent specimens. As shown in Figure 5b and Table 11, the locations of NIR fluorescence signals and corresponding auditory triggers were over 99.8% consistent, demonstrating the high reliability and robustness of the proposed system.

This system is not merely an incremental enhancement that converts visual information into auditory signals but rather represents a new paradigm that bypasses the physical visibility limits of conventional NIR imaging and directly extracts photon-level information [44]. While camera-based imaging fundamentally fails in strong tissue scattering environments, the high-sensitivity photosensor employed in this system enables signal detection even within the diffusive regime by directly measuring the absolute photon flux, as illustrated in Figure 5b.

To reliably capture ultra-weak fluorescence signals (<0.5 μW), a UV-bonded sealing structure and optimized black housing-based optical shielding were implemented, resulting in a 66% reduction in background noise and a 4.77 dB improvement in SNR. This represents a critical achievement that converts previously undetectable signals into quantitative and clinically meaningful data.

Furthermore, the system functions as a pre-visual guidance tool by detecting occult lymph nodes missed by conventional cameras with a high recall rate (98.4%). Through auditory feedback, it significantly reduces cognitive switching costs and visual fatigue associated with traditional vision-based navigation systems.

Existing near-infrared (NIR) camera-based methods [40,47] are analogous to observing a distant light source through dense fog, as illustrated in Figure 5c. As the thickness of adipose tissue surrounding the lymph node increases, fluorescence emission undergoes strong multiple scattering, leading to spatial diffusion of the signal. Consequently, the lymph node boundary becomes blurred and indistinguishable from surrounding adipose tissue, resulting in a low-contrast, diffuse image on the monitor.

This phenomenon occurs because NIR photons lose their directionality due to multiple scattering as they propagate through biological tissue, eventually entering the diffuse regime. In this regime, fluorescence signals become indistinguishable from background noise, fundamentally limiting image-based fluorescence detection beyond a few millimeters of tissue depth [54].

To overcome this physical limitation in image formation, this study employed a proximity-based energy detection method using a photosensor. The proposed approach captures the minute photon flux immediately above the lymph node before significant scattering occurs and converts ultra-weak fluorescence energy (<0.5 μW), which is invisible to conventional cameras, into real-time acoustic signals for accurate localization. Unlike camera-based methods that require a high signal-to-background ratio (SBR) for image reconstruction, this system measures only the absolute photon energy, enabling detection even under severe visual occlusion caused by adipose tissue.

To reliably detect such ultra-weak signals, background noise was minimized through structural optical sealing and controlled surgical environmental conditions. Instead of introducing bulky shielding structures that could interfere with surgical workflow, UV bonding-based sealing and a black housing design were implemented to maximize optical isolation. As a result, the system enables an auditory-based hybrid guidance framework that simultaneously mitigates visual overload and reduces cognitive switching costs associated with conventional vision-dependent navigation.

The core contribution of this study lies in three fundamental differences compared to conventional camera-based imaging and monitoring approaches. First, the proposed system identifies lymph node locations using auditory alarms, enabling real-time localization and quantification with significantly reduced identification time. Second, quantitative evaluation demonstrates high consistency between sensor-based lymph node detection and conventional NIR camera-based monitoring, confirming experimental accuracy and reproducibility.

During fluorescence-guided lymph node detection, both blood vessels and lymph nodes are stained, which may lead to ambiguity in photodetector-based alarms, as shown in Figure 5d. However, since the primary clinical risk is failing to detect lymph nodes, this study focuses on maximizing sensitivity rather than prematurely filtering vascular signals. Future work will address this limitation by distinguishing dynamic fluorescence fluctuations caused by blood flow from stable fluorescence signals originating from fixed lymphatic tissues.

To enable clinical translation, the system is being developed for integration into laparoscopic camera units via trocar docking. This requires miniaturization of the sensor module using semiconductor fabrication techniques, and follow-up research is currently underway. While this initial study validated feasibility using ex vivo specimens, future work will focus on structural optimization and long-term stability verification for in vivo applications. The ultimate goal is to develop an integrated device compatible with standard laparoscopic camera systems or trocar platforms.

Furthermore, future research will integrate photosensor alarm data with NIR camera images using a CNN-based algorithm to establish an audio–visual cross-validation surgical guidance system. As shown in Figure 5e, the proposed photosensor-based system reduced lymph node detection time by approximately 60% compared to conventional visual NIR fluorescence methods (Table 12 and Table 13), demonstrating its potential for efficient and reliable intraoperative navigation.

While conventional methods require repeated visual monitoring of an external display, the proposed system significantly improves navigation efficiency by providing immediate spatial information through auditory feedback. These results demonstrate that the system can reliably detect and convert fluorescence signals into sound, independent of individual anatomical variations, indicating high reliability and robustness.

The sound-based detector proposed in this study was designed to simultaneously address the physical limitations of conventional NIR imaging systems and the issue of visual fatigue experienced by surgeons. First, although fluorescence-based techniques may stain not only lymph nodes but also blood vessels, potentially leading to false-positive alarms [50], the more critical clinical risk is failing to detect lymph nodes altogether [47]. The proposed system prioritizes sensitivity, enabling the detection of extremely weak signals even in deep tissue where optical penetration is limited [55]. In this sense, the system functions as an ultra-sensitive detector that minimizes the probability of missed lesions. By providing an initial auditory cue before visual confirmation, the system allows surgeons to preemptively localize suspected lymph node regions and approach them more efficiently. Subsequent visual inspection can then distinguish between blood vessels and true lymph nodes, thereby creating a complementary multimodal guidance strategy. Although direct quantitative evidence regarding the reduction in total surgical time remains limited [56], it is evident that the proposed system substantially reduces cognitive load and visual distraction caused by repeated attention shifts between the surgical field and external monitors. Moreover, given its high probability of detecting occult lymph nodes [52], the system is expected to reduce unnecessary exploration time and improve overall surgical efficiency. Because lymph nodes are often visually indistinguishable from surrounding adipose and connective tissues in terms of color and texture, it remains extremely challenging for surgeons to identify them intraoperatively without technical assistance. In particular, indocyanine green (ICG)-based fluorescence guidance, which is widely used in breast cancer and melanoma surgeries, still presents several inherent technical limitations. First, the physical properties of near-infrared (NIR) light restrict its effective tissue penetration depth to only a few millimeters to approximately 1 cm, which can result in inaccurate detection of deeply located sentinel lymph nodes [40,47,50]. Second, strong light scattering within biological tissue blurs fluorescence boundaries, thereby hindering precise localization of metastatic lesions [47]. Third, the spatiotemporal separation caused by repeatedly shifting attention between the surgical field and an external monitor can induce visual overload and disrupt surgical workflow [56].

Metastasis to regional lymph nodes (LNs) is a well-established prognostic factor in colorectal cancer. The decision to administer adjuvant chemotherapy after surgery is largely based on the number of LN metastases. Micrometastases in LNs occur in approximately 5–6% of cases, as summarized in Table 14. Several studies report that LN micrometastasis is a significant prognostic indicator of systemic spread of occult cancer, given that up to 25% of node-negative colorectal cancer patients ultimately die due to tumor recurrence [57].

Lymph nodes (LNs) vary in size, making them difficult to identify when small or visually indistinguishable, particularly in patients with higher adipose tissue distribution. Currently, manual dissection, or the palpation method, is widely used for LN retrieval in surgical specimens. This approach relies on direct visual inspection and palpation by the surgeon or pathologist, and remains largely unstandardized, with practices differing across countries.

The increasing number of specimens further limits the feasibility of manual methods. LN retrieval through manual dissection is time-consuming, highly dependent on the experience and skill of the practitioner, and can affect diagnostic outcomes. Additionally, this process can delay test results by approximately a week, prolonging uncertainty for patients. Therefore, there is a critical need for improved methods that enable standardized, efficient, and reproducible LN retrieval, reducing both procedural time and variability in operator skill.

**Figure 6:**
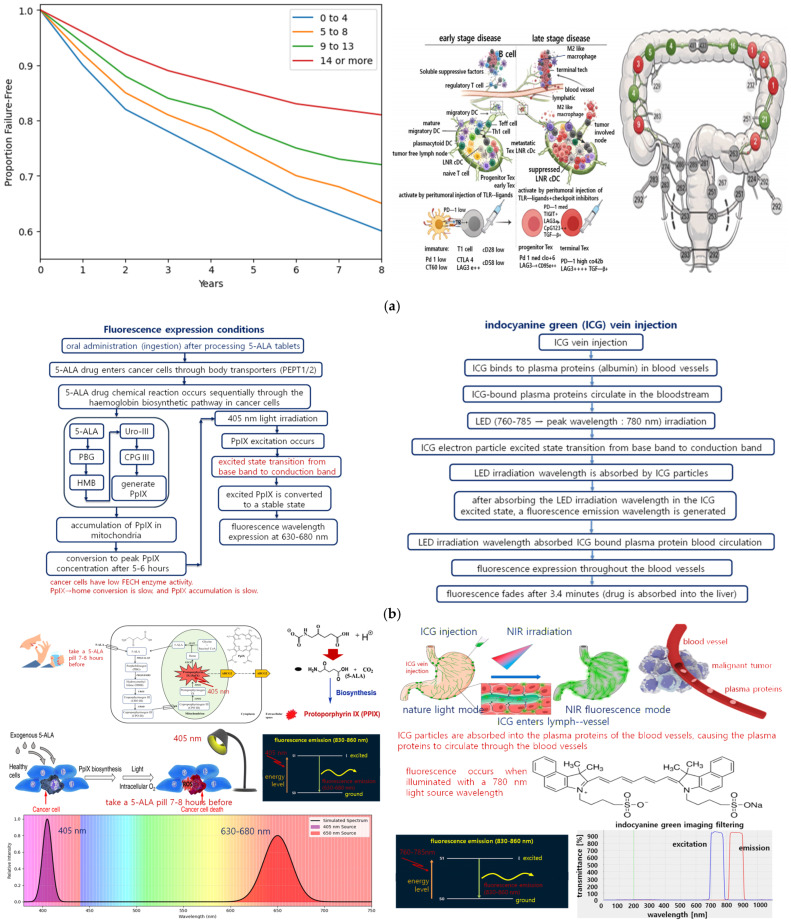

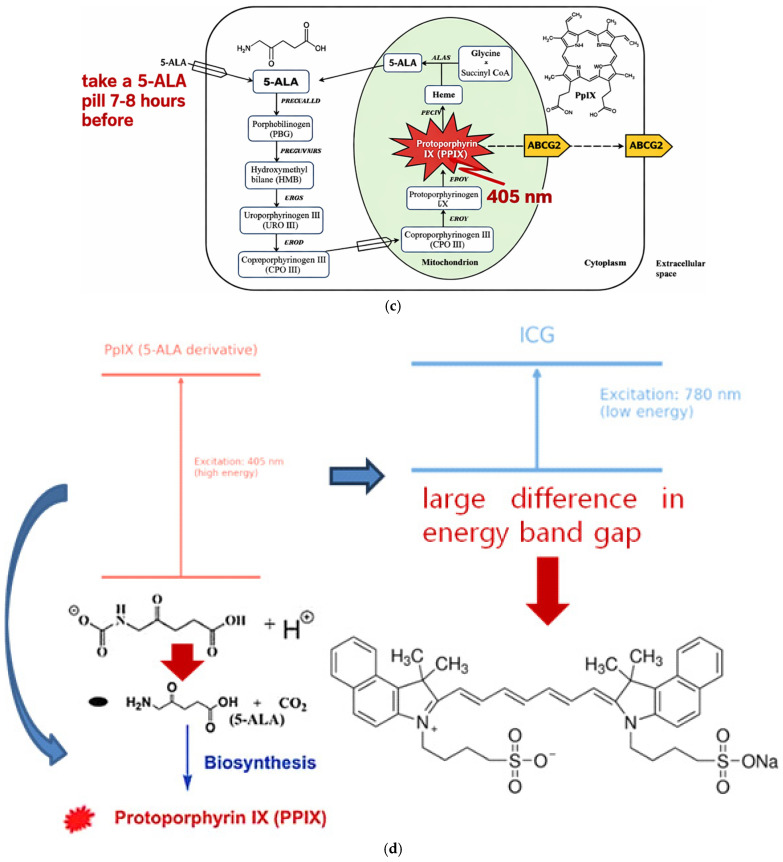
Clinical relevance of lymph node assessment and fluorescence mechanisms: (**a**) impact of tumor-draining lymph node evaluation on rectal cancer prognosis, showing (**left**) recurrence-free survival of N0 rectal cancer patients according to quartiles of examined lymph node counts and (**right**) schematic illustration of immune cell interactions and T-cell exhaustion in tumor-draining lymph nodes by disease stage, (**b**) fluorescence emission process of 5-ALA, (**c**) fluorescence emission process of ICG, and (**d**) comparison of fluorescence emission mechanisms and chemical structures between 5-ALA and ICG.

Effective LN harvesting techniques and standardization are essential not only for clinical accuracy but also for enhancing hospital competitiveness on both domestic and international levels. Consequently, a fluorescence imaging camera system that enables real-time differentiation and observation of tumor-involved LNs is highly desirable. Simultaneously, detection of fluorescence wavelengths using a photo sensor is crucial. As shown in Figure 6a, the periphery of the tumor is surrounded by blood vessels and adipose tissue, the latter exhibiting intrinsic autofluorescence above 800 nm [58]. In camera-based monitoring, this adipose autofluorescence, coupled with its color similarity to LN fluorescence emission, can mask the target signal. Incorporating a photo sensor allows for dual detection and improves reliability in identifying lymph nodes.

Assuming the results of this study are applied to future clinical settings following clinical trials and medical device approval procedures, this paper is expected to serve as a practical reference for medical device developers and clinical experts, such as hospital practitioners. In particular, given that documentation for medical device approval requires citations and technical evidence of device performance, the optical performance evaluation metrics and experimental validation results presented here can serve as useful supporting data. Accordingly, this review discusses the clinical and regulatory applicability of the proposed system, reflecting key requirements typically demanded during the medical device approval process.

To ensure clinical suitability, usability evaluations should be conducted as part of the medical device approval procedure. Additionally, if further research and development are required for regulatory approval, the topics presented in this review can serve as a reference for researchers seeking to advance similar medical devices. This review, therefore, focuses on analyzing observed phenomena and discussing the significance of the results, providing foundational data for device approval.

Clinically, fluorescent contrast agents are primarily classified into those used for tumor visualization and those used to observe blood flow in vessels. Malignant tumors are highly vascularized, and the colors of tumors and surrounding vessels are often similar, making visual differentiation challenging. This can lead to accidental vessel damage during tumor excision. Conversely, limiting tumor removal to protect vessels may result in incomplete resection. Since malignant tumors are highly invasive and prone to metastasis, incomplete resection significantly increases the risk of recurrence within five years.

Consequently, 5-aminolevulinic acid (5-ALA) is used to visualize tumor location and resection status, while indocyanine green (ICG) is employed to assess vascular structure and blood flow. The combined use of 5-ALA and ICG enables clear visualization of tumor-vessel boundaries, making this approach highly advantageous in clinical practice. ICG is administered intravenously, whereas 5-ALA is taken orally as a pill. Upon exposure to an external light source, these agents undergo fluorescence excitation and emit light at their respective wavelengths.

For 5-ALA, orally ingested 5-ALA is metabolized into protoporphyrin IX (PpIX) over 5–8 h. When irradiated with a 405 nm LED or laser, the electrons in PpIX are excited from the valence band to the conduction band, resulting in fluorescence emission at 630–680 nm, as shown in Figure 6c [59,60,61]. Cancer cells exhibit low ferrochelatase (FECH) activity, leading to slower conversion of PpIX to heme and accumulation within tumor tissue. PpIX does not absorb light at wavelengths other than 405 nm, reflecting the fixed energy band gap required for electron excitation (Figure 6d).

ICG fluorescence occurs when intravenously injected ICG binds to plasma proteins, circulating in blood vessels and staining lymph nodes. Upon irradiation with light of 760–785 nm (peak 780 nm), electrons in ICG are excited and emit fluorescence at 830–860 nm. This emission enables the camera to capture spectral color corresponding to blood vessels and lymph nodes, allowing for differentiation on the monitor. The 830–860 nm band falls within the NIR region, where color perception is synthesized by the camera sensor. ICG does not absorb light outside 760–785 nm due to its fixed excitation band gap (Figure 6d).

Light source intensity must be sufficient to induce fluorescence. Lasers provide high intensity but narrow beam width and potential safety risks, whereas LEDs offer a wider field of view with lower intensity, requiring active intensity adjustment during surgery. While LEDs generate less heat and are safer for clinical use, fluorescence intensity may be lower, necessitating technical optimization.

Fluorescence-guided observation is applied variably depending on the surgical objective, patient characteristics, and tumor type. It may involve observing vascular flow, tumor location and resection status, or delineating tumor-vessel boundaries. Simultaneous use of 5-ALA and ICG is uncommon in practice; typically, each is used individually. However, if used concurrently, the distinct excitation and emission wavelengths, as well as differing electron excitation mechanisms, prevent interference, ensuring safe and effective visualization.

Fluorescence-guided observation is widely employed across surgical specialties, as well as in dentistry, radiation oncology, nuclear medicine, anesthesiology, pain medicine, and veterinary medicine, for visualizing tumors, lymph nodes, blood vessels, and inflammatory lesions. False alarms caused by vascular staining will be addressed in future work by distinguishing dynamic blood flow from static lymph nodes using a heartbeat-based signal analysis algorithm. Unlike ICG, which visualizes lymphatic flow, 5-ALA selectively accumulates in tumor cells themselves. Beyond cancer surgery, the proposed system can be extended to a wide range of fluorescence-guided applications, including angiography and lymphedema treatment.

## 5. Conclusions

This study proposes a method for detecting the presence and location of lymph nodes by administering a fluorescent contrast agent into the blood vessels of a specimen, thereby inducing fluorescence in the lymph nodes. Upon illumination with an LED, the lymph nodes emit fluorescent light at a characteristic wavelength, which is detected by a photosensor. Detection of this fluorescence triggers an audible alarm via a speaker and activates an LED indicator, providing real-time guidance on the presence and location of lymph nodes. To evaluate the detection performance of the photosensor, a comparative analysis was conducted against near-infrared (NIR) camera imaging and monitoring results, enabling quantitative assessment of the system’s accuracy, reliability, and efficiency.

This study overcomes the limitations of existing technologies, which struggle to visualize deep tissue due to light scattering, by directly capturing photon flow. The ability to detect even extremely weak signals enables accurate identification of deeply concealed lymph nodes. While further research is required to improve blood vessel differentiation and further reduce surgical time, the proposed technology demonstrates strong potential as a novel sound-based surgical guidance system that reduces surgeon visual fatigue and maximizes detection efficiency.

After detecting the fluorescent wavelength, the photosensor converts it into an electrical signal, which activates both the speaker and the LED to generate an audible alarm and visual indication. Given that the fluorescent emission power is extremely low (<0.5 μW), employing a high-efficiency sensor is critical for enhancing detection performance. To further improve detection efficiency, this study implemented an optical collection system combining an optical condenser with a convex lens. Detailed analysis of the wavelength-specific response characteristics and external quantum efficiency of the silicon PIN photodiode achieved a photoelectric conversion efficiency of approximately 80–90% within the 830–860 nm wavelength range. Additionally, bandpass filters were applied to both the LED and the photodetector, effectively blocking the overlap between the LED irradiation wavelength and the fluorescence wavelength. This configuration enabled the photodetector to selectively detect the fluorescence wavelength, thereby minimizing potential wavelength-based detection errors. Consequently, stable and reliable detection of the fluorescence signal was achieved. This study overcomes the physical limitations of NIR optical imaging through precise photon flow detection and optimization of multiple optical occlusion factors. The proposed system quantitatively demonstrated a 4.77 dB improvement in signal-to-noise ratio (SNR), enabling the detection of potentially obscured lymph nodes with high reproducibility (98.4%), even when visually concealed by fat tissue thicker than 5 mm. Furthermore, the introduction of auditory feedback reduces the cognitive load on surgical staff and suggests its potential as an eyes-free hybrid guidance tool that minimizes visual dependence. This approach holds clinical potential for improving microscopic lesion detection rates and shortening surgical time. Although future work is required to implement multiwavelength analysis and signal attenuation compensation to further enhance vascular differentiation, this study experimentally demonstrates the novel potential of an audio-based smart surgical guidance system. The proposed method is anticipated to substantially facilitate the rapid and accurate identification of lymph nodes in specimens obtained from patients undergoing colorectal cancer surgery.

## Figures and Tables

**Table 1:** Key performance analysis of photodiodes.

Parameter	Symbol	Typical Value	Unit	Note
detection wavelength	λ	400–1100	nm	fluorescence wavelength range (included 830~860 nm)
forward current	I_f_	5.0	mA	
reverse voltage	V_r_	30	V	
responsivity @ 850 nm	R_e_ (λ = 850 nm)	0.60	mA/mW	optimal sensitivity in the NIR wavelength region
operating temperature	T_op_	−40 to +85	°C	
responsivity @ 1064 nm	R_e_ (λ = 1064 nm)	0.20	mA/mW	sensitivity degradation onset region
active area	Φ	0.5 × 0.5	mm	active area
response time	T_r_	250	ps	high-speed response
dark current @ VR = 5 V	I_D_ (5 V)	50	pA	low-noise detection
reverse breakdown voltage	V_BR_	120	V	
junction capacitance @ V_R_ = 5 V	C_j_ (5 V)	1.5	pF	favorable for high-speed signal conversion
saturated optical power	P_s_ (5 V)	7	mW	
operating voltage	V_R_	0–20	V	
shunt resistance	R_sh_ (10 mV)	100	GΩ	maintains low-noise characteristics
package	–	hermetic TO46	–	compatible with filters and lenses

**Table 2:** Photodiode conversion efficiency and loss analysis at NIR fluorescence wavelengths.

Wavelength [nm]	Responsivity, R [A/W]	Maximum Rideal = qλ/hc [A/W]	Conversion Efficiency, η = R/Rideal [%] −10log10 (η)	Loss [dB] Loss = −10log10 (0.9)
830	0.60	0.67	0.60/0.67 = 90%	0.46 dB
850	0.58	0.68	0.58/0.68 = 85%	0.70 dB
860	0.55	0.69	0.55/0.69 = 80%	0.97 dB

**Table 3:** Conversion efficiency and corresponding loss ratio in the NIR region.

Wavelength [nm]	Efficiency, η [%]	Loss Ratio [× Times]
830	90.0	1.11
850	85.0	1.18
860	80.0	1.25

**Table 4:** Key performance analysis of the filter (FL830-10)—substrate material: schott borofloat, coating: dielectric thin films (T_i_O_2_/S_i_O_2_).

Transmission Wavelength (λT) [nm]	FWHM [nm]	Minimum Transmittance (T) [%]	Optical Density (OD) [%]	Cut-On Slope [nm]	Size (Diameter) [mm]
830	10 ± 2 nm	70	≥4.0	3 to 70	25.4 mm

**Table 5:** Transmission loss and loss ratio of optical filter at 830 nm [33,34].

Performance	Value	Calculation
Transmission loss	30%	(1 − 0.70) × 100% = 30%
loss ratio	1.43 times	loss ratio = 1/0.70 = 1.4286
dB loss	1.55 dB	dB loss = −10log10 (0.70) = 1.55 dB
OD (optical density)	≥4.0	OD = −log10 (T), T = 4.0^−4^

**Table 6:** Key performance analysis of optical condenser (ACL25432U).

Transmission Wavelength [nm]	Focusing Distance (f) [mm]	Numerical Aperture (NA)	Materials	Coating	Diameter [mm]	Thickness Thin/Height [mm]
830 nm	32.0	0.10	B270 Schott (glass)	uncoated (380–2100 nm)	25.4	14.0

**Table 7:** Key performance analysis of convex lens (LA1951-AB-ML-N-BK7, Thorlabs, Inc., Newton, NJ 07860, USA).

Transmission Wavelength [nm]	Focusing Distance (f) [mm]	Materials		Diameter [mm]	Thickness Thin/Height [mm]
		Substrate	Coating		
400–1100	25.4	N-BK7 (glass)	anti-reflection (AR)	25.4	1.8/11.7

**Table 8:** Key performance analysis of optical condenser (ACL25432U) and Convex Lens (LA1951-AB-ML).

Performance	ACL25432U	LA1951-AB-ML
fluorescence wavelength (λ) [nm]	830	400–1100
focal length (f) [mm]	32.0	25.4
numerical aperture (NA)	0.10	0.10
material	B270 Schott (glass)	N-BK7 (glass)
coating	uncoated	AR coated
diameter [mm]	25.4	25.4
thickness [mm]	14	1.8/11.7

**Table 9:** Electrical output characteristics after photodetector.

Stage	Current (mA)	Voltage (V)	Waveform	Characteristic
alarm	0.04	2.03	DC	voice turn on
LED	0.08	4.47	DC	LED turn on
photodetector (DMM)	0.12	4.81	DC	fluorescence detection

**Table 10:** Comparison of current NIR imaging technology with the proposed method.

Comparison Item	Current and Advanced Technologies Imaging and AV/AI	Proposed Technology Auditory Sensor System	References
operating principle	2D image reconstruction and AI pattern recognition	direct capture of photon energy at the source energy flux	[40,47,48]
data dependency	dependent on input image quality	direct extraction of raw physical signals	[48]
physical limitation	signal loss within the diffusive regime	overcoming diffusion limits via proximity scanning	[49,50]
noise handling	risk of misinterpretation by AI due to image noise	UV sealed structure and illumination control achieving 4.77 dB SNR	[51,52]
perceptual modality	visual AID based AR monitor with cognitive load	auditory feedback eyes free guidance	[53]
clinical novelty	software centered navigation through image optimization	hardware sensing paradigm focused on signal recovery	[48,49,52,54]

**Table 11:** Consistency analysis between NIR fluorescence imaging and auditory sensor detection.

Specimen No.	Number of Lymph Nodes (n)	NIR Detection (Positive)	Auditory Detection (Positive)	Concordance Rate (%)
specimen 1	12	12	12	100%
specimen 2	15	15	15	100%
specimen 3	11	11	11	100%
specimen 4	13	13	13	100%
specimen 5	14	14	13	92.8%
total	65	65	64	98.5% (avg.)

**Table 12:** Performance comparison between the conventional visual method and the proposed auditory-guided system (↔ Display for observing the specimen by connecting it to a monitor).

Parameters	Conventional Visual Monitoring (NIR Only)	Auditory-Guided Feedback (Proposed)	Improvement/Result
mean detection time (s/node)	5.0–10.0 s	2.0–4.0 s	~60.0% reduction
operator fatigue (gaze dispersion)	high (specimen ↔ monitor)	low (auditory trigger)	-
spatial correlation (with NIR imaging)	N/A	100% alignment	-
total identified lymph nodes (n)	-	62 nodes (5 specimens)	-

**Table 13:** Quantitative performance comparison of lymph node detection methods.

Parameters	Conventional Visual Monitoring	Auditory-Guided Feedback (Proposed)	Improvement
mean detection time (s)	7.5 ± 1.8	3.0 ± 0.6	~60.0%
detection time range (s)	5.0–10.0	2.0–4.0	-
total nodes evaluated (n)	62	62	-
spatial consistency (with NIR image)	N/A	100%	-
note: data were obtained from 5 surgical specimens (ex vivo).			

**Table 14:** Clinical outcomes of n_0_ rectal cancer patients by number of examined lymph nodes [57].

Number of Examined Lymph Nodes	Number of Patients (n)	5-Year Relapse Rate (%)	5-Year Survival Rate (%)
0–4	127	37	68
5–8	138	34	73
9–13	129	26	72
≥14	133	19	82

## Data Availability

Data are contained within the article and Supplementary Materials.

## Supplementary Materials

The following supporting information can be downloaded at: https://www.mdpi.com/article/10.3390/s26061745/s1, Figure S1: Structure and fabrication process of the proposed optical detection module; Figure S2: Optical transmission loss minimization of the proposed optical module (simulation and experimental validation); Figure S3: Experimental setup configuration and phantom fluorescence example; Figure S4: Photocell performance test environment using a fluorescent phantom; Figure S5: Photocell performance test results for fluorescent wavelength detection; Figure S6: NIR camera imaging results of the fluorescence emission phantom; Table S1: Quantitative analysis of gap-size-dependent stray light loss and SNR recovery after UV bonding.

## Abbreviations

**abbreviation**	**definition**
ICG	indocyanine green
NIR	near-infrared
SNR	signal-to-noise ratio
FGS	fluorescence-guided surgery
CV	computer vision
AI	artificial intelligence
UV	ultraviolet
LED	light emitting diode
