# Supplementary material for: A Novel Fluorescence-Triggered Auditory Feedback Photosensor for Precision Lymph Node Mapping
Source: Sensors (Basel). 2026 Mar 10;26(6):1745. doi: 10.3390/s26061745 (PMC13030055; doi:10.3390/s26061745)
Supplement: Supplementary file 1 [file sensors-26-01745-s001.zip › sensors-4151922-supplementary.pdf]

Supplementary Note S1 : Design, fabrication, and optical loss analysis of the proposed photo sensor

As shown in Figure S1(a), the proposed structure integrates multiple lenses (optical filter, convex lens, and condenser) with a photodiode. The attachment was realized using 3D printing technology, as illustrated in Figure S1(b), and the components were assembled in the following order: optical condenser, optical filter, convex lens, and photodiode, as depicted in Figure S1(c). The metal wire-based additive manufacturing process used for fabricating the module is schematically shown in Figure S1(a) and Figure S1(b). The sensor module frame was fabricated using fused deposition modeling (FDM) 3D printing technology. The laminated structure was optimized to ensure precise alignment of the internal optical components within a tolerance of 0.1 mm.

In the metal wire-based additive manufacturing process, aluminum wire is supplied to a melting chamber and locally heated by a pulsed current applied to a coil winding. The molten metal is then ejected through a nozzle by gravity and pressure differences, while argon gas is injected to prevent oxidation and impurity contamination. The ejected material is deposited onto a preheated substrate, and a computer-controlled motion system regulates the movement of the substrate or nozzle to achieve layer-by-layer deposition, ultimately forming the three-dimensional structure.

When optical components such as the condenser lens, optical filter, convex lens, and photodiode are assembled, ambient light can infiltrate the gaps between the components, leading to performance degradation. To suppress this effect, UV adhesive was injected into all inter-component gaps, as shown in Figure S1(c). The assembled module was then inserted into a cylindrical frame fabricated by 3D printing using black PLA filament, which encloses the components and provides optical shielding.

The silicon PIN photodiode exhibited a high photoelectric conversion efficiency of approximately 80% in the 830–860 nm wavelength band. Filling the gaps with UV adhesive is crucial, as it prevents light refraction and loss caused by air layers, thereby increasing the signal-to-noise ratio (SNR) and blocking external stray light. The reflection loss at the air–glass interface was theoretically estimated to be approximately 4%, and the simulation results were consistent with this value, confirming the validity of the proposed design. The black filament used for the outer casing absorbs near-infrared light, suppresses internal reflections, and minimizes interference from external illumination, thereby further enhancing system performance.

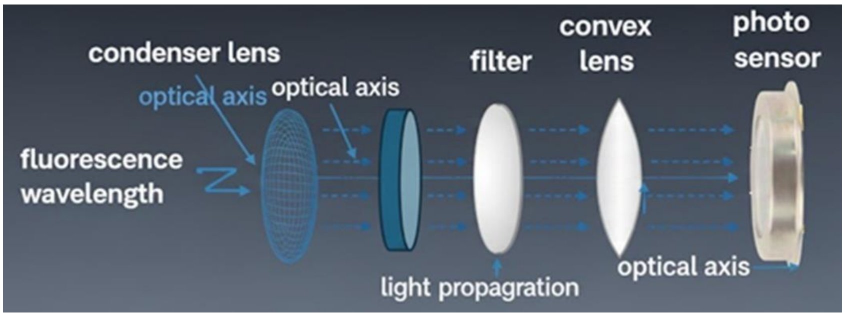

(a)

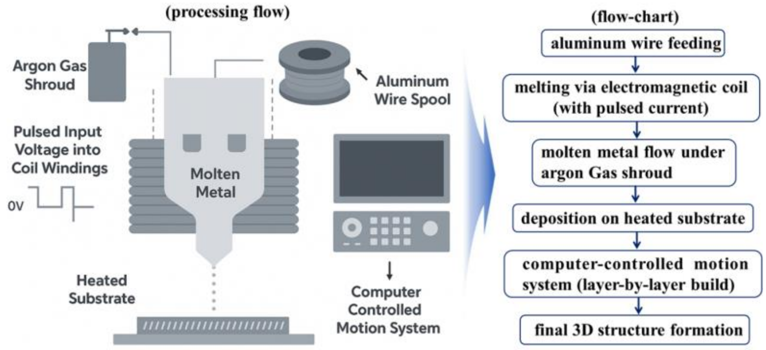

(b)

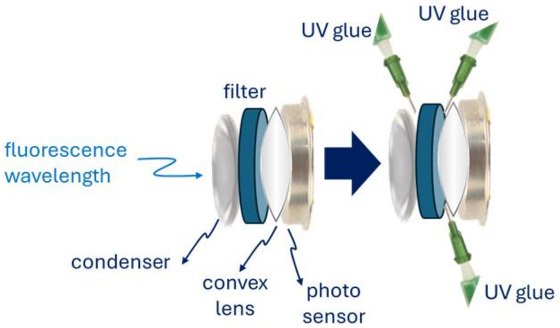

(c)

Figure S1. Structure and fabrication process of the proposed optical detection module (a) schematic of the proposed structure with a lens attached to the photodiode (b) module fabrication and assembly process using 3D printing technology (c) assembly sequence of the optical condenser, optical filter, convex lens, and photodiode, along with the light-blocking structure using UV adhesive

In a structure combining an optical condenser, optical filter, and convex lens with a photosensor, the reflection and transmission characteristics of light passing through each interface can be analyzed using Fresnel's law (Equation (SE-1)), where R, T, η1, and η2 denote the reflectance, transmittance, refractive index of the incident medium, and refractive index of the transmitted medium, respectively. The values of T, η1, and η2 were set to 95.7%, 1.0, and 1.52, respectively.

The theoretically calculated reflection loss (R) was estimated to be approximately 4.25%, as shown in Table TS-1, which is primarily attributed to the refractive index mismatch at the air–glass interface [36]–[39]. However, based on the simulation results of the coupled structure presented in Figure S1(a), the actual reflection loss was below 4.0%. Nevertheless, as shown in Figure S1(c), approximately 4% of external light enters through the coupling gap and can act as an interfering component by overlapping with the fluorescent emission. Therefore, this loss is non-negligible compared to the filter transmission loss (≈30%) or the photosensor's photoelectric conversion loss (≈0.5–1.0 dB) [36]–[39].

$$R=\left[\frac{\eta_1-\eta_2}{\eta_1+\eta_2}\right]^2 \quad @ \; T = 1 - R \qquad \text{(SE-1)}$$

Table TS-1. Quantitative analysis of gap-size-dependent stray light loss and performance recovery after UV bonding. UV adhesive filling significantly reduces effective optical loss and improves signal-to-noise ratio (SNR), particularly for larger inter-component gaps [36]-[39]

gap size [µm]	lost optical power before UV [µW/cm²]	loss ratio before UV [%]	stray light loss before UV [%]	pptical power increase after UV [%]	recovered optical power after UV [µW/cm²]	effective loss after UV [%]	SNR improvement after UV [dB]
50	0.35	17.5	17.5	60	+0.21	2.1	+1.9
100	0.45	22.5	22.5	67	+0.30	2.3	+2.5
200	0.60	30.0	30.0	73	+0.44	2.6	+3.2
300	0.75	37.5	37.5	80	+0.60	2.9	+3.9
500	0.90	45.0	45.0	82	+0.74	3.2	+4.6

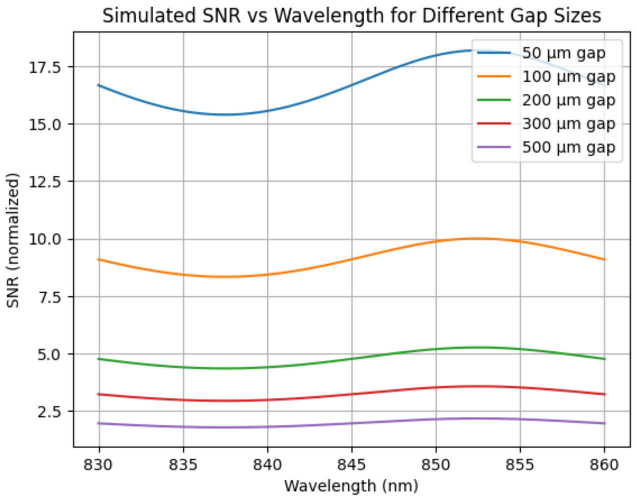

(before)

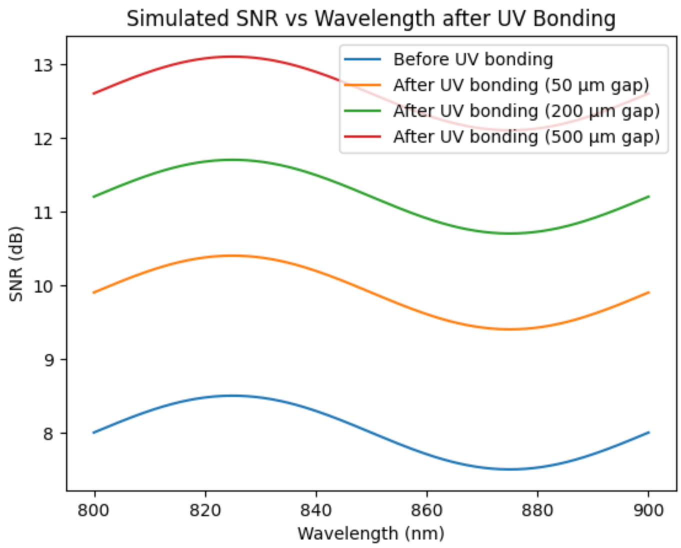

(after)

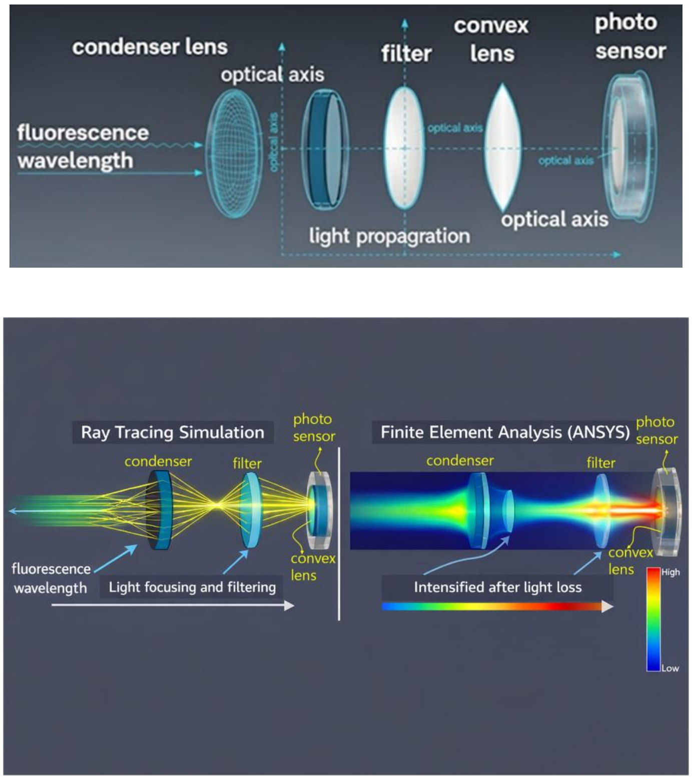

(design structure)

(a)

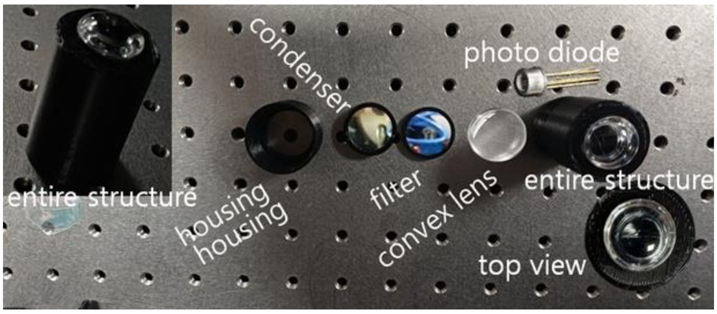

(b)

Figure S2. Optical transmission loss minimization of the proposed optical module: simulation and experimental implementation. (a) simulated signal-to-noise ratio (SNR) versus wavelength for various inter-component gap sizes before and after UV bonding, together with the optical module design generated using Tinkercad (model name, company name, city, country). (b) photograph of the fabricated and assembled optical detection module.

The results of minimizing the gap between the optical lenses using UV bonding, as shown in Figure S2(a), revealed a significant improvement in SNR. This indicates that the proposed structure provides an environment in which Fresnel reflections at the air–glass interface are reduced, stray light entry paths are effectively blocked, and the effective light reception power is increased. Consequently, the SNR improved by several decibels, and the overall SNR deviation was significantly reduced. When manufacturing the outer case via 3D printing, a black filament material was used to block light transmission and absorb unnecessary external light sources. Figure S2(b) shows the actual design and a photograph of the manufactured structure. The overall dimensions are approximately 65 mm in length, 45 mm in diameter, and 25.4 mm in bore diameter.

Supplementary Note S2 : experimental environment setup and phantom test

The experimental setup for lymph node detection using a photosensor is configured as shown in Figure S3(a). It consists of an LED light source capable of exciting a fluorescent target (M780L3, Thorlabs, Inc., Newton, New Jersey, USA), an NIR camera (LT225 NIR M/N, Thorlabs, Inc., Newton, New Jersey, USA), and a photodetector (TO46 PIN photodiode, NaKu Technology Co. Ltd., Hangzhou, Zhejiang, China).

The NIR camera is connected to an external monitor. The photodetector is connected to a signal measurement device (oscilloscope, TBS1052B, Tektronix, Danaher Corporation, Beaverton, OR, USA) to evaluate fluorescence detection performance, and to a digital multimeter (DMM, Fluke Corporation, Everett, WA, USA), a speaker (Arduino SZH-SSBH-007, NTREX, Incheon, Republic of Korea), and an LED (Arduino F5 5 mm green LED, NTREX, Incheon, Republic of Korea) to provide audible and visual alerts indicating fluorescence emission.

All signals from the photosensor, alarm, LED, and voltage monitoring system are processed and controlled via a microcontroller board (Arduino Nano, Arduino AG, NTREX, Incheon, Republic of Korea).

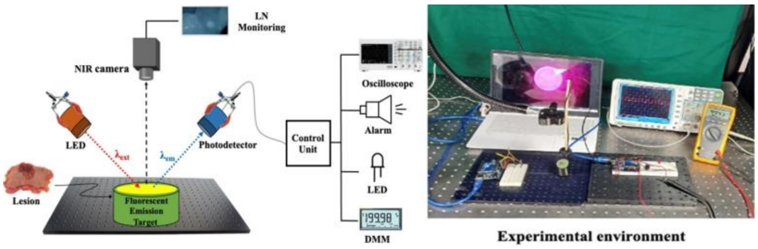

(a)

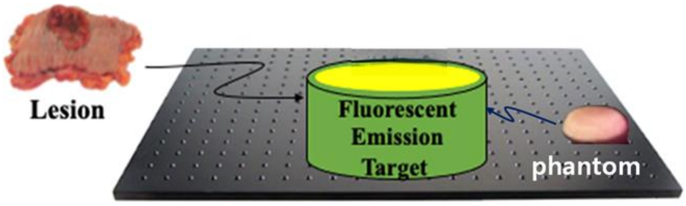

(b)

Figure S3. Experimental setup configuration (a) phantom environment configuration (b) phantom fluorescence emission example

To obtain experimental results, two experimental approaches were employed. The first approach involved experiments using a fluorescence-emitting phantom, as shown in Figure S3(b) [41]. When the phantom was illuminated with an LED light source, fluorescent wavelengths were emitted. These signals were captured by an NIR camera and displayed on an external monitor. Simultaneously, the photosensor detected the emitted fluorescence and generated multiple outputs, including electrical signals, voltage readings, LED indicators, and auditory alarms.

The second approach involved injecting a fluorescent contrast agent into biological specimens to achieve fluorescent labeling of lymph nodes. Upon LED illumination, the lymph nodes emitted fluorescent wavelengths. These emissions were visualized using the NIR camera and displayed on the monitor. In parallel, the photosensor detected the fluorescence and produced corresponding outputs, including electrical signals, voltage levels, LED activation, and auditory alarms.

The overall experimental configuration for evaluating fluorescence wavelength detection performance is shown in Figure S4. The system comprised a fluorescent phantom, LED light source, NIR camera, external monitor, photosensor, oscilloscope, speaker, LED, and digital multimeter (DMM), all integrated on a dedicated test board.

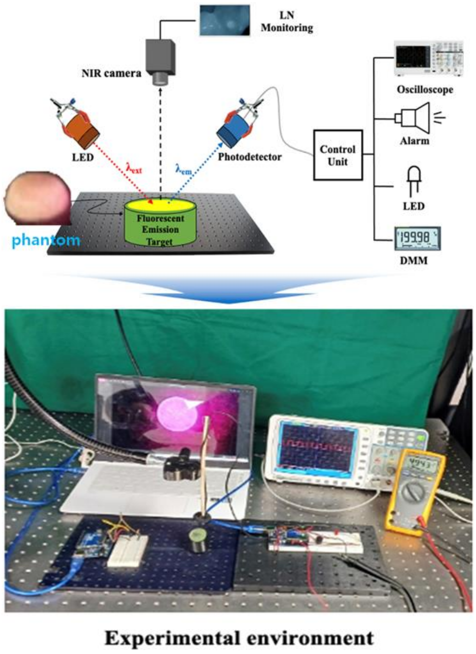

Figure S4. Photocell performance test environment setup using a fluorescent phantom

When an LED with an irradiation wavelength of 760–785 nm was directed at a fluorescence phantom designed to emit near-infrared signals (emission range: 805–860 nm; model name, company name, city, country), the phantom generated fluorescence in the 830–860 nm range, as shown in Figure S5. Because the LED was equipped with a bandpass filter, only wavelengths between 780 and 785 nm reached the phantom, resulting in selective excitation of the fluorescent emission band.

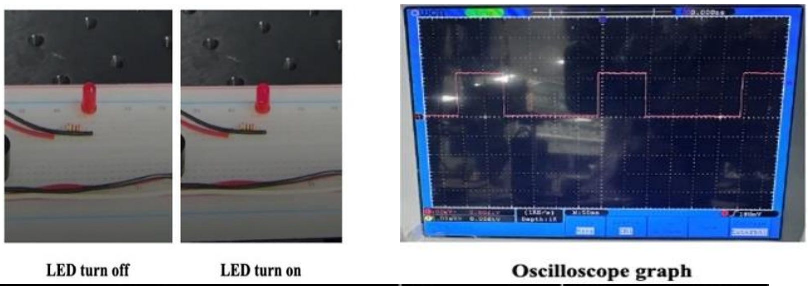

Performance		Parameter	Characteristic
Alarm	Current [mA]	0.04	Voice turn on
	Voltage [V]	2.03	
	Wave Form	DC	
LED	Current [mA]	0.08	LED turn on
	Voltage [V]	4.47	
	Wave Form	DC	
DMM	Voltage [V]	4.81	Fluorescence detection

Figure S5. Photocell performance test results for fluorescent wavel ength detection using a phantom

The photosensor has a wide spectral detection range (400–1100 nm), enabling it to detect both the LED excitation wavelength and the fluorescence emission wavelength. Therefore, a bandpass filter (830 ± 2 nm, FL830-10, Thorlabs, Inc., Newton, NJ, USA) was employed to selectively transmit only the fluorescence band (830–860 nm). Consequently, the photosensor detected only the emitted fluorescence within this range. The optical signal was converted into an electrical signal via the control board, yielding the results shown in Figure S5.

The results can be categorized into conditions before and after fluorescence detection. Before fluorescence detection, the LED was turned off, and no signal was observed on the oscilloscope. The DMM indicated 0 V, and no alarm was generated, with measured current and voltage values of 0.013 mA and 0.02 V, respectively. Under these conditions, the NIR camera image shown in Figure 10 exhibited a dark background, making it difficult to visually identify the phantom.

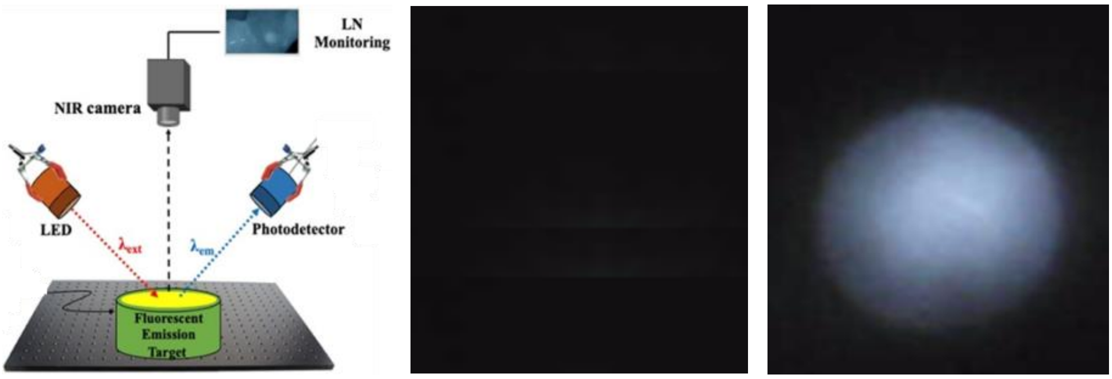

(fluorescent phantom experiment) (before fluorescent emission) (after fluorescent emission)

Figure S6. NIR camera imaging results of the fluorescence emission phantom before and after

When the LED irradiates the phantom and the photosensor detects the fluorescence wavelength, the indicator LED is activated, generating a pulse signal on the oscilloscope. In addition, the DMM records an output voltage of 4.81 V, an alarm is triggered, and the speaker circuit produces a signal with a current of 0.04 mA and a voltage amplitude of 2.03 V, as measured by the DMM and oscilloscope. Consequently, as shown in Figure S6, the monitoring video captured by the NIR camera displays only the fluorescent phantom, while the surrounding background remains dark.
